# Polymeric Materials in Speciation Analysis Based on Solid-Phase Extraction

**DOI:** 10.3390/molecules29010187

**Published:** 2023-12-28

**Authors:** Ivanka Dakova, Tanya Yordanova, Irina Karadjova

**Affiliations:** Faculty of Chemistry and Pharmacy, University of Sofia “St. Kliment Ohridski”, 1, James. Bourchier Blvd.1, 1164 Sofia, Bulgaria; i.dakova@chem.uni-sofia.bg (I.D.); t.yordanova@chem.uni-sofia.bg (T.Y.)

**Keywords:** polymeric materials, speciation, chemical elements, solid-phase extraction

## Abstract

Speciation analysis is a relevant topic since the (eco)toxicity, bioavailability, bio (geo)chemical cycles, and mobility of a given element depend on its chemical forms (oxidation state, organic ligands, etc.). The reliability of analytical results for chemical species of elements depends mostly on the maintaining of their stability during the sample pretreatment step and on the selectivity of further separation step. Solid-phase extraction (SPE) is a matter of choice as the most suitable and widely used procedure for both enrichment of chemical species of elements and their separation. The features of sorbent material are of great importance to ensure extraction efficiency from one side and selectivity from the other side of the SPE procedure. This review presents an update on the application of polymeric materials in solid-phase extraction used in nonchromatographic methods for speciation analysis.

## 1. Introduction

Speciation analysis, defined as an analytical procedure for the identification of chemical species of trace elements existing in the sample and their quantification, is probably the most actual analytical task. According to the IUPAC (International Union of Pure and Applied Chemistry) statement “chemical species” is the “specific form of an element defined as to isotopic composition, electronic or oxidation state, and/or complex or molecular structure” and element chemical speciation means its “distribution amongst defined chemical species in a system” [1,2]. The analytical process for speciation analysis of solid samples conventionally consists of the extraction of chemical species, avoiding any reactions between them leading to their change, further their separation by appropriate technique, followed by sensitive instrumental measurement. As expected for the speciation analysis of liquid samples, the extraction step is not necessary. Although the reliability of the results obtained depends on the performance of each stage of the procedure, the selectivity of the separation of chemical forms is of utmost importance. Combining the separation with the next instrumental measurement is no less essential. Hyphenated methods coupling online separation steps with sensitive instrumental measurement are widely used for speciation analysis [3,4], although as cheaper alternatives, nonchromatographic approaches are also frequently applied for selective determination of highly toxic species [5,6,7]. In both cases, the processes of sorption and desorption of target species determine the accuracy and reliability of the results obtained. For hyphenated methods, the choice of chromatographic separation depends on the physical properties of the target species. For volatile species, e.g., for Hg speciation, gas chromatographic methods are a preferable technique [8]. For nonvolatile analytes, various types of liquid chromatography are widely used [9]. Excellent review papers discussed approaches, types of packing materials, and their applications for the speciation of Hg, As, Se, Sn, etc. [9,10,11,12,13,14]. In nonchromatographic methods, solid-phase extraction (SPE) is the most common approach used for the enrichment and selective separation of chemical forms of elements in nonchromatographic methods. The advantages of SPE are well known and thoroughly discussed in the literature—the possibility of performing simultaneous enrichment and separation of chemical species, almost no consumption of toxic solvents, easy development of online methods combining separation steps with sensitive detection, high potential for automation, etc. Different types of techniques were used to improve the efficiency, selectivity and practicality of solid-phase extraction for speciation analysis of chemical elements. Magnetic SPE gains applicability due to its very good practicality, avoiding tedious centrifugation or filtration of sorbent by using its magnetic properties, which allow easy removal from the sample medium by an external magnetic field [15,16]. Solid-phase microextraction (SPME) is highly popular due to the low consumption of samples and reagents, thus responding to the principle of green chemistry [17,18]. In the field of nonchromatographic speciation analysis based on SPE, the high selectivity of sorbent materials has a strong impact on the reliability and accuracy of the whole analytical procedure, and for this reason, ion-imprinted materials are highly preferable. In recent years, nanosized materials and composites containing nanoparticles have been widely used due to their highly active surface and reactivity toward selected species [19,20]. Polymeric sorbents, often referred to as polymer-based adsorbents or resin adsorbents, are materials composed of synthetic or natural polymers that are widely used for all kinds of solid-phase extractions due to the possibility for highly selective retention of particular chemical species based on the specific functional groups, incorporated in the polymer matrix. Polymeric sorbents are typically made from natural polymers such as chitosan, cellulose, β-cyclodextrin, dextran or synthetic polymers such as polystyrene, polyacrylate, polyethylene, or others. The choice of polymer depends on the specific application and the target compounds to be adsorbed. The polymeric material plays a crucial role in analytical and separation processes because the extraction efficiency and selectivity strongly depend on the active functional groups. Due to the huge variety of structures and functionalities of the polymers, they offer many opportunities for development of reliable procedures for speciation analysis. In all cases, an important requirement is to avoid any interactions with sorbent particles leading to the changes of chemical species of studied analytes.

**Figure 1:**
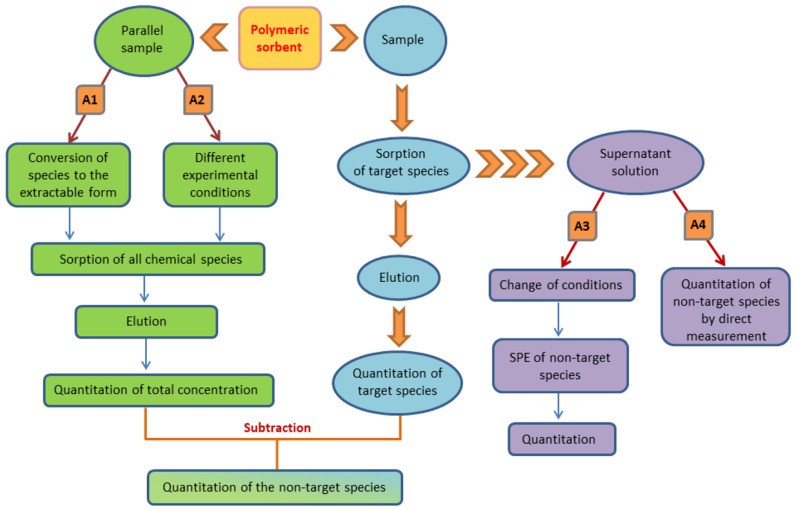
Common approaches in SPE-based speciation analysis assisted by polymeric materials.

From an analytical point of view, SPE-based speciation procedures using polymeric sorbents have been carried out according to several basic protocols described in Figure 1. The most common approach (A1) is based on the selective extraction of the target chemical form combined with a parallel conversion of the coexisting species to the extractable one. In this way, the target species and total content of the element are quantified in the eluate solutions after SPE, and the concentration of nonextractable species is usually obtained as the difference between these two measurements. In the following discussions of this review, this approach will be referred to as Approach 1 (A1). Another option using a parallel sample is to run the SPE under different conditions, e.g., pH, ensuring the sorption of all species (A2). A possible way to determine two (or more) chemical forms in a sample is the successive extraction of particular species accomplished by changing conditions (A3). But if highly sensitive measurement instruments, e.g., inductively coupled plasma mass spectrometry (ICP-MS), electrothermal atomic absorption spectrometry (ETAAS), or atomic fluorescence spectrometry (AFS), are used, then the nonextractable species can be directly quantified in the supernatant solution after retention of the target analyte (A4).

This review provides an updated summary of the most important features and applications of polymeric materials used for column packing in nonchromatographic strategies as efficient and selective sorbents for the last 15 years. Most of the articles are from the period 2016–2023, about 60% of total, and between them, about 50% are from the last years, 2020–2023. Several papers published before 2016, which we assessed as important pieces of the development of polymeric materials as sorbents, are included, and these are less than 10%. Recent achievements in the field of solid-phase microextraction, magnetic solid-phase extraction, and uses of ion-imprinted polymers for speciation analysis of different kinds of samples will be discussed, and future perspectives will be outlined.

## 2. Polymeric Materials for Solid-Phase Extraction

### 2.1. Commercially Available Polymeric Materials Applied as Sorbents

The commercially available traditional polymeric sorbents such as Amberlite, Dowex, Lewawit, etc., are found to be very promising sorbents for SPE of metal ions due to their good physical and chemical properties, such as porosity, high surface area, durability and purity [21]. As a rule, their selectivity is low since the sorption mechanism is based on electrostatic attraction and ion exchange. However, they can be easily modified by chemical bonding or impregnation with chelating agents and, in this way, characterized by better selectivity.

#### 2.1.1. Commercial Polymeric Anion Exchangers

The commercially available anion exchange resin Amberlite IRA 900 was used for the selective sorption of As(V) at pH 4, both arsenic species were retained at pH 8, and finally, As(III) was determined by subtractions. Electrothermal AAS was used for measurements using Ni chemical modifier [22]. The analytical procedure was validated by the analysis of certified reference material (CRM) and applied for the speciation of arsenic in hot ground water and tap water. A similar procedure using minicolumn filled with strong anion exchanger styrene (ST), divinylbenzene (DVB), and ethylstyrene-containing polymer gel functionalized with the chloride form of quaternized trimethylamine was applied for the speciation and further removal of arsenic from drinking water and geothermal water [23,24]. Hosseini et al. described a method for arsenic speciation analysis in water samples following strategy A1 in which preconcentration of arsenic as As(V) was coupled with spectrofluorometric determination [25]. The speciation of As was realized using a column packed with strong anion exchange resin (Amberlite IRA-410). The negatively charged As(V) was selectively retained on the column in the presence of As(III). Total As was determined after As(III) oxidation with H_2_O_2_. Detection of As(V) was performed spectrofluorimetrically in the presence of L-cysteine-capped-CdS nanoparticles. The proposed method has many advantages, such as accuracy, simplicity, high analytical throughput, good limit of detection (LOD) and low maintenance costs.

An analytical scheme for As(III)/As(V) separation and speciation was realized by using a novel sequential injection system incorporating two minicolumns followed by detection with hydride generation atomic fluorescence spectrometry (HG-AFS) [26]. An octadecyl immobilized silica minicolumn is used for selective retention of the complex between As(III) and ammonium pyrrolidinedithiocarbamate (APDC), while the sorption of As(V) is readily accomplished by an anion exchange resin minicolumn. The retained As(III)–PDC complex and As(V) are effectively eluted with a 3.0 M HCl solution, which well facilitates the ensuing hydride generation process via reaction with tetrahydroborate. Acharya et al. analyzed ground water samples for total arsenic and its inorganic species contents by instrumental neutron activation analysis (INAA) [27]. Two separation methods using the strong anion exchange resin Dowex 1X8 in an acetate form were developed using radiotracers for As(III)/As(V). In the first method, the selective elution of both arsenic species retained from 8 M HCl was achieved with 8 M acetic acid. In the second selective retention, As(V) was done from 8 M acetic acid, further eluted with 0.12 M HCl. The second method, based on strategy approach 4, was applied for As speciation in ground water. Similar to the first described method, it was applied to determine total arsenic, As(III) and both inorganic As species in ground water using charged particle activation analysis (CPAA) (~16 MeV proton beam) [28]. The DOWEX 1X8 resin is in acetate form; loading and eluting solutions are 0.1 M HCl.

An interesting approach with sufficiently low detection limits was proposed for the determination of inorganic arsenic species (iAs) in water samples by coupling the borohydride form of the anion exchanger with the HG-AFS [29]. The method is based on the generation of arsine (AsH_3_) from the reaction between the arsenic species in the injected solution and tetrahydroborate immobilized on a strong anion exchange resin (Amberlite IRA-400). The specification scheme was based on strategy approach 2 with two different measurement conditions: (i) acidification to 0.7 M HCl, and (ii) acidification to 0.1 M HCl in the presence of 0.5% L-cysteine.

Three types of resins have been studied for the separation of iAs and organic As (oAs) species—a commercially available gel-type strong base anion exchange (SBAE) resin Lewatit Mono-Plus M 500, (styrene-divinylbenzene copolymer), a hybrid macroporous monodispersed polystyrene-containing resin based on the activity of hydrated iron oxides (Fe-resin), and homemade silver loaded ion exchange resin (Ag resin) [30]. Results obtained showed selective separation of four As species by using strong base anion exchange resin—As(V), monomethyl arsenic acid (MMAs), dimethyl arsenic acid (DMAs) are retained on the resin, while nonsorbed neutral specie As(III) could be determined in the effluent after sorption. All arsenic species except DMAs(V) were sorbed on the Fe resin, which ensures selective determination of this specie in the effluent. All inorganic As species were retained on Ag resin, which ensures the selective separation of inorganic and organic As species. Calculated resin capacities showed good tolerance toward the potential interference of anionic compounds. An analytical procedure was developed for As speciation in drinking, natural, and wastewater using ICP-MS or HGAAS for As measurement. A similar method is proposed for the separation and determination of only inorganic As species in natural and drinking water [31]. Two resins, a SBAE resin and a hybrid (HY) resin, were utilized. Separation of As(III) and As(V) species on the SBAE resin was accomplished by adjusting the acidity of water samples to a pH value less than 8.00; the ionic forms of As(V) were retained while the molecular forms of As(III) remained in the water. Enrichment of both inorganic As species was accomplished with the HY resin. Arsenic was analyzed by ICP-MS.

The anionic ion exchange resins, in combination with chelating resin packed in a column, are incorporated in an online system coupled to ICP-MS for Cr speciation [32]. Both species, in this case, are determined separately after selective retention of chromate on the Amberlite IRA 910 anion exchanger and Cr(III) on the chelate resin, in this way avoiding the use of any additional reagents and subtraction of results. Relatively low detection limits were found at 0.009 μg/L for Cr(VI) and 0.03 μg/L for Cr(III), and the method was applied for the determination of Cr species in seawater.

Saçmaci et al. developed a method for Cr(III)/Cr(VI) speciation in various environmental samples based on SPE combined with flame atomic absorption spectrometry (FAAS) following strategy scheme A1. Total chromium was measured by FAAS [33]. Lewatit Ionac SR-7 anion exchange resin (a strong basic quaternary ammonium salt) was used for the selective separation and preconcentration of Cr(VI) anions. The main advantages of the proposed method are its high sorption capacity (17.2 mg/g), high enrichment factor (500), and low LOD (3 ng/L). It is also fairly rapid, low cost, and uses fewer chemicals. The ion exchange method using the sulfate form of Dowex-1 (strong anion exchanger) was shown to be suitable for the speciation of Cr(III) and Cr(VI) in sediment and soil samples [34]. The Cr species were extracted from samples using a solution containing 0.1 M EDTA, 1% tetrabutyl ammonium bromide and a little HF in a domestic microwave oven. The quantitative sorption of the Cr(VI) was achieved at pH 4.5. The anion of Cr(III), i.e., Cr[EDTA]^−^, present in the extract, did not adsorb onto the resin. The determination of the separated Cr(VI) and total Cr in the extracts, as well as the total Cr after total digestion, was carried out using ICP-OES. The accuracy of the proposed method was checked with a CRM, stream sediment (GBW-07312). A similar procedure was developed by the same team for chromium speciation in samples from black tea, green tea, spinach, and fruit trees [35].

Gredilla et al. proposed a simple and nonexpensive analytical scheme for the effective separation of Hg(II) and methylHg in hydrochloric or chloride media based on the strategy scheme A4 using the weakly basic anion exchange resin Dowex M-41 [36]. Under defined chemical conditions, the negatively charged HgCl_4_^2−^ is retained by anionic exchangers, while the noncharged CH_3_HgCl passes through the resin with negligible retention and is measured in the effluent. In this way, both Hg species are determined in a single injection of sample.

A simple, sensitive, and selective method was developed for the determination of Sb(III) and Sb(V) in meglumine antimoniate using the selective separation of Sb(III) and ICP-OES detection following strategy approach A4 [37]. The separation of Sb(III) was achieved after selective retention of its chloro-complexes on an SBAE resin (Dowex 1X4), enabling Sb(III) separation in the presence of a high concentration of Sb(V).

#### 2.1.2. Commercial Polymeric Cation Exchangers

Unusual cation exchange was also used for As speciation. The in situ separation of As(III) and As(V) was performed after the addition of APDC to the sample and retention of As(III)–PDC complex on the upper PTFE miniature disk while As(V) was retained on the lower Zr and Ca loaded cation exchange disk [38]. Arsenic on both disks was measured directly by wavelength X-ray fluorescence (XRF) spectrometry after their fixation to an acrylic plate using adhesive cellophane tape. The method was applied for As speciation in spring water and well water. Widely used reagent diphenylcarbazide for selective spectrophotometric determination of Cr(VI) was used in a column SPE for selective Cr speciation using strategy approach A1 [39]. The complex of Cr(VI) with diphenylcarbazide was electrostatically sorbed on commercial cation exchange resin TSK IC-Cation placed in a minicolumn and eluted by a mixed solution containing lanthanum chloride and 1-propanol. The minicolumn was incorporated into a high-performance liquid chromatography (HPLC) system with UV detection. The determination of total Cr was achieved in a parallel sample after oxidation by low-pressure UV lamp (185 nm). The analytical procedure was characterized and used for Cr speciation in water samples. Speciation and preconcentration of Cr(III) and Cr(VI) were performed using strongly acidic Amberlite CG-120 resin before the detection step by FAAS following again strategic scheme 1 [40]. Cr(III) was adsorbed in a column, while Cr(VI) was not under optimal SPE conditions. Total chromium was determined after Cr(VI) reduction to Cr(III).

Table 1 presents an overview of the reported sorbents and analytical procedures for the separation and speciation of chemical elements using commercially available polymeric materials.

#### 2.1.3. Modified and Impregnated Commercial Resins

An aminated Amberlite XAD-4 resin beads filled in a column incorporated in Flow Injection Cold Vapor Generation Atomic Absorption Spectrometry (FI-CVG-AAS) system was tested as a solid-phase extractant for Hg speciation [42]. The separation of Hg was based on the retention of both Hg(II) and methylHg on the aminated Amberlite XAD resin beads followed by selective elution for Hg(II) with thiourea in HCl and for methylHg with 6 M HCl. After method validation (CRM), it was successfully applied to the analysis of fish and mussel samples. Aminated Amberlite XAD-4 resin was used as an efficient sorbent for the preconcentration and speciation of Cr(III) and Cr(VI) ions by column technique [43]. Selective retention of Cr(III) ions was achieved at pH 8.0 and eluted using 1.0 mL of 3.0 M HCl and 1.0 mL of 2.0 M NaOH, successively, at the flow rate of 5.0 mL/min. The maximal sorption capacity of the resin for Cr(III) ions was found to be 67.0 mg/g. The developed method was successfully applied for chromium speciation in mineral, drinking and wastewater samples with low LOD, high enrichment factor, good accuracy and repeatability. Simple flow injection FAAS method for Cr speciation in industrial water was developed using selective adsorption of Cr(III) on chelating resins prepared by chemical immobilization of 1,10-phenanthroline, xylenol orange, α-benzoin oxime and salicylic acid on Amberlite XAD-16 [44,45,46,47]. Total Cr was determined after reduction of Cr(VI) to Cr(III) using hydroxylamine hydrochloride. The accuracy of the method was confirmed by the analysis of CRM. The same team of authors developed an analogous procedure for the Cr specification using Dowex Optipore L493 modified with dithizone [48].

Narin et al. developed a speciation procedure for chromium(III), chromium(VI) and total chromium in environmental samples based on adsorption of Cr(III)–diphenylcarbazone complex on Amberlite XAD-1180 resin prior to FAAS determination [49]. Cr(III)–diphenylcarbazone complex was prepared after reaction between diphenylcarbazide and Cr(VI) in sulfuric acid medium (0.5 M). Cr(III) ions under these conditions were not recovered. After oxidation of Cr(III), the developed solid-phase extraction system was applied to determine the total chromium. Cr(III) was calculated as the difference between the total Cr content and the Cr(VI) content. The procedure was successfully applied to the environmental and pharmaceutical samples.

A simple, facile and economic procedure for Cr speciation and preconcentration was developed using Dowex M 4195 chelating resin (macroporous divinylbenzene copolymer with the bis-picolylamine functional group) [50]. This sorbent selectively retained Cr(VI) anions at pH 2. The total chromium was determined after oxidation of Cr(III) to Cr(VI) by using H_2_O_2_. The concentration of Cr(III) was calculated by the difference between total chromium and Cr(VI) concentration. The presented method was applied for the Cr speciation in natural water samples with satisfactory results (recoveries > 95%, RSDs < 10%).

An accurate, precise and simple method was developed for speciation and determination of Mn(II) and Mn(VII) ions in water samples utilizing a macroporous resin, Amberlite XAD-7HP and FAAS measurements [51]. Amberlite XAD-7HP resin has been shown to retain Mn(VII) between pH 4 and 12. Mn(II) determination can be carried out by forming the MnO_2_ precipitate at pH 12. The elution from the microcolumn and/or the dissolution of MnO_2_ formed can be realized by K_2_C_2_O_4_ in HNO_3_, which has been shown to be very effective. Quantitative recoveries (≥96%) with industrial wastewater samples showed the accuracy and applicability of the presented method. New sorbent material prepared from Amberlite XAD-1180 modified with ionic liquid CYPHOS 101—trihexyl(tetradecyl)phosphonium chloride) was used as a “portable kit” for the on-site speciation procedure for the determination of total mercury, Hg(II), and methylHg [52,53]. The sorbent-packed cartridge selectively retained Hg(II) while the methylHg passed without significant sorption. The Hg(II) on the sorbent was recovered with 6 M HNO_3_. Mercury species were determined using square wave anodic stripping voltammetry with a solid gold electrode and using a portable potentiostat. The technique might be used on-site.

A controlled porous structure and adjustable surface area are well-known advantages of polymeric sorbents. In addition, when negative and positive charges are located in close proximity on the surface of the sorbent, this offers better extraction efficiency over anion or cation exchange sorbents only. Polymer zwitterion microspheres have been synthesized based on Merrifield resin with a cationic core, modification with 1,2-ethanedithiol (S-version) or imidazole (N-version), and an outer shell containing an anionic sulfonic group [54]. Both sorbents packed in a homemade minicolumn showed high affinity toward Hg(II), methylHg, and ethylHg and can be used for quantitative enrichment of three Hg species before the hyphenated system HPLC-ICP-MS using a ZORBAX SB-C18 column. Experiments performed indicated that the absorption efficiency for N-version is higher than that of S-version. A column packed with N-version was used for Hg species enrichment in surface and seawater.

The analytical procedures proposed for chemical element speciation using modified and impregnated commercial resins are summarized in Table 2.

### 2.2. Polymeric Sorbents Prepared with Additional Functionalization

In order to improve selectivity, polymer resins with various modifications have been proposed. Additional functional groups incorporated on the surface of polymeric material ensured, in most cases, a sorption mechanism based on chelate formation. An interesting idea for selective sorption of As(V) is the development of chemisorbent (ImpAs) based on the incorporation of a metal–organic complex containing two Zn(II) centers [56] in the polymeric beads [57]. The combination of handheld syringes filled with polymeric beads with anodic stripping voltammetry leads to a portable analytical tool for arsenic speciation. The possibility to separate in one run the four arsenic species (As(III), As(V), MMAs, DMAs) is achieved by using a phosphine-modified polymer microsphere filled with a homemade minicolumn [58]. The large number of positively charged adsorption groups ensures quantitative retention of all four arsenic species, which, after fast elution, are directed to the hyphenated HPLC-ICP-MS system. The results obtained are validated against commercially available Hamilton PRP-X100 columns and CRM analysis. One of the easiest approaches is to use a preliminarily prepared chelate complex of the analyte, which will be retained on the polymer sorbent. This approach was followed by Döker et al. As(III) as PDC complex was retained on poly(hydroxyethyl methacrylate), and As(V) passed through the column [59]. Total arsenic was determined after the reduction of As(V) by thiourea. Arsenic was measured by ETAAS. The method was validated by the analysis of CRM and applied to inorganic As speciation in drinking water and snow samples.

The FAAS method for Cr speciation in industrial water was developed using selective adsorption of Cr(III) on 1,10-Phenanthroline immobilized Amberlite XAD-16 chelating resin [44]. Total Cr was determined after the reduction of Cr(VI) to Cr(III) using hydroxylamine hydrochloride. The accuracy of the method was confirmed by the analysis of CRM. A similar approach was used for the speciation of Cr in water and food samples using different homemade chelating resins: poly(N,N′-dipropionitrile methacrylamide-co-divinylbenzene-co-2-acrylamido-2-methyl-1-propanesulfonic acid) resin [60], poly-2-(5-methylisoxazole)methacrylamide-co-2-acrylamido-2-methyl-1-propanesulfonic acid-co-divinylbenzene) [61]. Şahan et al. proposed a new online approach for Cr speciation in which the chelating resin (poly 2-(5-methylisoxazol)methacrylamide-co-2-acrylamido-2-methyl-1-propanesulfonic acid-co-divinylbenzene) was used in combination with a strong anion exchange resin both placed in a minicolumn [62]. Both Cr species are retained on the sorbent in the column and further sequentially eluted and determined by FAAS. In this way, the addition step of reducing Cr(VI) to Cr(III) is avoided. A relatively complicated synthesis procedure was used for the preparation of sorbent selective toward Cr(VI)-poly(styrene-divinylbenzene) microbeads were graft-coated with poly(oligo (ethylene glycol) methacrylate)-block-poly(glycidyl methacrylate) and further modified with phosphomethylated triethylene tetramine [63]. In this case, total chromium was determined after oxidation of Cr(III) with permanganate. The same approach was also proposed by Hazer et al. using poly(1,3-thiazol-2-yl methacrylamide-co-4-vinyl pyridine-co-divinylbenzene) as chelating resin for Cr(VI) [41]. In-laboratory synthesized anion exchange sorbent N,N-bis(2-aminoethyl)ethane-1,2-diamine functionalized poly(chloromethyl styrene-co-styrene) was used only for the enrichment of both Cr species [64]. The retention of cationic Cr(III) was ensured after the addition of EDTA to form an anionic [Cr(III)–EDTA]^−^ complex. The speciation of Cr in wastewater was achieved after fast elution with tetrabutylammonium hydroxide and measurement by hyphenated HPLC-ICP-MS. An analytical approach based on the combination of an iminodiacetate extraction disk placed on a cation exchange extraction disk for Cr(III) and an anion exchange extraction disk for Cr(VI) was developed and applied for Cr speciation using metal furnace AAS (metal furnace was used as an atomizer) [65]. The method was used for Cr speciation in river water, and a discussion on the stability of Cr(VI) in such systems is presented. Tokalıoğlu et al. [66] synthesized a new sulfonamide-containing polymer (5ATT-CSPS) using chemical modification of chlorosulfonated polystyrene resin with 5-amino-1,3,4-thiadiazole-2-thiol. The new sorbent 5ATT-CSPS was applied for chromium speciation in various water samples and in lettuce extracts obtained in the unified bioaccessibility method for saliva by dispersive SPME (d-SPµE). The total chromium concentration was determined by FAAS. It was found that the Cr(III) ions were quantitatively adsorbed in the range of pH 2.0–4.0, while Cr(VI) was not adsorbed in this pH range and its concentration was determined indirectly. The enrichment factor of the method was found to be 8, the limit of detection—2.9 μg/L, and RSD—≤4.3%. A relatively simple speciation procedure was developed for Hg(II) and methylHg separation using poly(acrylamide) grafted onto cross-linked poly(4-vinyl pyridine) [67]. Batch experiments showed that Hg(II) is quantitatively sorbed on the sorbent while methylHg remains quantitatively in the solution. In this way, an analytical procedure was developed for Hg speciation using measurements by CV AFS and applied to seawater and estuarine water analysis. A polymeric material very specific toward Hg species was synthesized by reaction between monomers of the vinyl derivative of 8-hydroxyquinoline (8-HQ) and 2-(Methacryloylamino) ethyl 2-Methyl Acrylate [68]. Both Hg(II) and methylHg were retained on the sorbent placed in a glass solid-phase extraction cartridge. Separation of Hg species was achieved by using selective eluents 2 M HCl in methanol for Hg(II) and strong oxidizing agent NaClO for methylHg, and further detection was easily achieved by highly sensitive AFS. The accuracy of the approach was confirmed by analyzing CRM, and finally, an analytical procedure was developed for Hg speciation in fish samples.

The selective retention of Sb(III) in the presence of Sb(V) on a polyurethane foam loaded with bromopyrogallol red was used for Sb speciation in water samples [69]. The amount of Sb(III) was quantified by ETAAS in the eluate solution (2.5 M nitric acid). The concentration of Sb(V) was determined in the efluate after Sb(III) selective removal. The method was applied for Sb speciation in river water and antihistamine drug samples A three-column system was accordingly designed, using the two adsorbents in tandem, for the separation and enrichment of the antimony species [70]. The method was developed on the basis of selective sorption of Sb(III) on a chelating resin [1,5-bis(2-pyridyl)-3-sulfophenyl methylene] thiocarbonohydrazide immobilized on aminopropyl-controlled pore glass. After the removal of Sb(III), Sb(V) was collected on Amberlite. The accuracy of the proposed method for Sb speciation was confirmed with two CRMs: SLRS-5 River Water and TMDA-54.4 Fortified Lake Water. The values obtained were in accordance with those reported for the certified materials. The method was applied for seawater analysis.

### 2.3. Polymer-Supported Ionic Liquid

The poly(ionic liquid)s (PILs, also called ionic liquid-based polymer gels or ionogels) are “polyelectrolytes that comprise a polymeric backbone and an ionic liquid (IL) species in monomer repeating units” [71]. Unlike classical polyelectrolytes, which are water-soluble and dissociate in aqueous solutions to give charged polymers, most PILs are insoluble in water, making them suitable materials for sorbents in SPE [18,72]. Moreover, PILs possess high stability (thermal, chemical and mechanical) and various functional groups. By choosing a suitable type of IL immobilized in the polymer matrix, it is possible to achieve high adsorption capacity, extraction efficiency and selectivity towards different classes of analytes, including inorganic species.

A novel online solid-phase extraction approach for the separation and preconcentration of Cr(VI) is developed in a sequential injection system with two minicolumns [73]. One minicolumn selectively retains Cr(VI) by bonding onto the positively charged surface of PIL (poly(vinyl chloride) functionalized with 1-chlorovinyl-3-methylimidazolium chloride). The sorbed Cr(VI) was effectively recovered by elution with 0.2 M NH_4_NO_3_ solution, and the eluate was quantified by ETAAS. The coexisting Cr(III) is pre-eliminated by a strong acidic cation exchange resin minicolumn. Chromium speciation is performed by measuring the amount of Cr(VI) and total chromium after converting Cr(III) into Cr(VI) via oxidation.

Thangaraj et al. proposed a new PIL material with potential use for the separation of Sb(III) and Sb(V) for analytical estimations of the oxidation states of Sb in environmental samples [74,75]. The sorbent was synthesized by cross-linking copolymerization of 1-butyl-3-vinylimidazolium bromide with N, N-methylene diacrylamide. Antimony binding properties of a PIL are investigated in the presence of complexing organic acids. Sb(III) specific binding against Sb(V) was found in the presence of nitrilotriacetic acid.

Ionic liquids, an alternative to regular solvents, have been immobilized on different solid supports as a part of “green chemistry” and applied as effective sorbents for trace elements. Inorganic Hg as an anionic chlorocomplex was quantitatively retained on a sorbent prepared by the impregnation of Amberlite XAD-1180 resin with an ionic liquid tetradecyl(trihexyl)phosphonium chloride filled in a homemade column, while, under the same conditions, the sorption of methylHg was negligible [55]. The retained iHg was simultaneously eluted and reduced with 5% (*w*/*v*) SnCl_2_ in 2.4 M HCl and directed into the gas–liquid separator of CV-AAS instrument for quantification. Total Hg content was determined by the same procedure after methylHg photo-oxidation under a UV lamp. The analytical procedure was used for Hg speciation in various water samples.

### 2.4. Magnetic Polymeric Materials for Solid-Phase Extraction

The process of separating sorbent from sample media is a tedious step that usually requires long centrifugation or filtration, long-lasting, especially in the case of nanocomposites or nanoparticles. The incorporation of magnetic core in the sorbent material solves such problems and magnetic polymeric sorbents are proposed and widely used for efficient SPE procedures. Efficient As speciation was achieved by using multifunctionalized polydopamine-coated magnetic graphene [76]. The sorbent was used as column packing material where both As species were retained simultaneously and further separated by selective elution. An analytical procedure developed was used for As speciation in natural and environmental water samples. Well-known sorbent magnetite coated with polythiophene was tested for Cr speciation in a microsample injection system FAAS [77]. It was found that Cr(III) is selectively retained in the presence of Cr(VI) and an analytical approach was developed for the determination of Cr(III), total Cr (after reduction of Cr(VI) and Cr(VI) by subtraction. The method was validated by analyzing several CRM-Tibet soil, strawberry leaves and applying it to Cr speciation in food, biological, and soil samples. A new sorbent was prepared following a relatively complicated synthesis procedure: Macroporous magnetic poly (glycidyl methacrylate-co-ethylene glycol dimethacrylate) is obtained by in situ suspension copolymerization in the presence of Fe_3_O_4_ nanoparticles coated with a silanization agent 3-aminopropyltriethoxysilane (APTES) and further functionalized with diethylenetriamine with the main aim of selective removal of Cr(VI) [78]. The results indicated that Cr(VI) oxyanions that bind to the sorption sites can be converted on the surface of the sorbent to a less harmful form of Cr(III) due to the sorbent’s chemical composition. An effective analytical method was designed by synthesizing sorbent, combining the advantages of porous organic polymers with magnetic nanoparticles and incorporating this sorbent in a hyphenated method HPLC ICP-MS [16,79]. Two types of magnetic organic porous polymers were prepared following almost identical schemes. The synthesis procedure consists of several steps—synthesis of magnetic nanoparticles and monomer followed by polymerization process between them to obtain a magnetic organic porous polymer. Finally, thiol–ene “click” reaction was performed for the post-modification of polymer with 1,2-ethanedithiol. A magnetic porous organic polymer modified with thiol groups demonstrated high extraction efficiency toward Hg(II), methylHg and phenylmercury (phenylHg) in [16] or Hg(II), methylHg, ethylmercury (ethylHg) and phenylHg in [79]. After sorption, polymer particles are easily removed from the sample solution and transferred in a tube where all Hg species are eluted with thiourea in HCl, and the eluent was introduced into HPLC ICP-MS for detection. The analytical method was validated and applied for Hg speciation in rice, soil, water and fish samples. A novel magnetic ionic liquid nanocomposite was synthesized and applied for the selective retention of Te(IV) species, followed by elution with 5 M HCl and determination by FI-HG-AFS [80]. Strategy scheme 1 was used, and the total Te was quantified after a prereduction step. The developed magnetic dispersive microsolid-phase extraction was successfully applied for the determination of Te(IV) and Te(VI) species in environmental samples with different levels of matrix complexity, including natural water, soils and sediments. Selective sorption of only inorganic Se(IV) and Te(IV) was demonstrated using polyaniline functionalized MNPs [81]. The analytical procedure proposed included ICP-MS measurement of retained species and was applied after validation with CRM for water samples analysis. Solid-phase microextraction was used for the enrichment of organic Sn species prior to HPLC measurement with fluorescent detection [82]. Magnetic nanoparticles were embedded in a porous polymer matrix in a capillary and utilized as the microextraction column for Sn species enrichment prior to HPLC separation. The method was applied for Sn speciation in seafood.

### 2.5. Polymeric Composites with Nanoparticles

Nanostructured materials have been regarded as excellent adsorbents for SPE. They are featured with a small-size effect, large surface area and plenty of unsaturated surface atoms, which could provide high adsorption capacity [19,20,83].

Efficient speciation of As was achieved with composite material—multiwalled carbon nanotube-branched polyethyleneimine packed in a minicolumn and detection by HG-AFS [84]. Selective sorption of As(V) was obtained at pH 5.8, while the sorption of As(III) was below 5%. Total As was determined after As(III) oxidation with hydrogen peroxide. The analytical procedure developed was validated by the analysis of CRM and applied for As speciation in rain and snow water. The same sorbent was further applied for selective enrichment and determination of As(V) using ICP-MS measurement [85]. The optimal pH for As(V) sorption in this case was 7. The results obtained for As(V) agreed well with the results obtained by hyphenated HPLC-ICP MS analysis. Nanocomposites were synthesized by carbon nanotube modification with methacrylic acid (MAA) and 4-vinylpyridine (4-VP) and packed in two minicolumns. The system was constructed in such a way that when the sorption of Cr(III) goes on one of the minicolumns, the desorption of Cr(VI) takes place on the other. The system permits sequential selective determination of Cr(III) and Cr(VI) measured by FAAS. The system was successfully used for Cr speciation in potable and mineral water [86]. Rapid and sensitive determination of toxic Cr(VI) was achieved utilizing highly fluorescent conjugated poly[(9,9-dioctylfluorenyl-2,7-diyl)-alt-co-(1,4-benzo-(2,1′,3)thiadiazole)] polymer dots [87]. Polymer dots were used for Cr(VI) quantification in water samples based on selective and significant fluorescent quenching. In addition polymer dots were functionalized by doping with isophthalic acid and studied for selective Cr(VI) tracking inside human cells.

### 2.6. Polymeric Materials for Solid-Phase Microextraction

Determination of both inorganic arsenic species was achieved by miniaturized solid-phase microextraction using polymer polystyrene polydimethyl siloxane loaded into the micropipette tip of the syringe system. Arsenic (V) was quantitatively retained while As(III) passed through the micropipette tip of the syringe system [88]. Total arsenic was measured after oxidation of As(III) with potassium permanganate and As(III) quantified by subtraction. A new sorbent, a derivative of the microbial polyester poly(3-hydroxy butyrate)—biodegradable poly-3-hydroxybutyrate-2-(dodecylthiocarbonothioylthio)-2-methylpropionate triester, was loaded in a micropipette tip and incorporated in a solid-phase microextraction system [89]. Chromium (III) is selectively adsorbed in the column, while Cr(VI) is not retained. Total Cr is determined after the reduction of Cr(VI) to Cr(III) hydroxy ammonium chloride. Electrothermal AAS was used for Cr measurement. This method is validated against CRM and applied for Cr speciation in water samples.

A simple method for Sb speciation was based on the selective sorption of Sb(III) as dithiocarbamate complex on the polystyrene oleic acid imidazole polymer at pH 5.5 [90]. The sorbent was placed in a micropipette tip of the syringe system. Quantitative elution was achieved with 2 M nitric acid, and Sb(III) in the eluate was measured by ETAAS. The total Sb was determined after the reduction of Sb(V) to Sb(III) with L-cysteine. An analytical procedure was used for Sb determination in water and food samples.

### 2.7. Ion-Imprinted Polymers as Sorbents for Speciation Analysis

Ion-imprinted polymers (IIPs) are synthetic materials possessing recognition sites able to specifically rebind a target ion. The high selectivity of IIPs toward the target metal ions is due to their memory effect that results from the preparation process [91]. IIPs are widely used as sorbents for SPE, stationary phases in chromatographic and injection columns, sensor development, and membrane separation [92]. Good thermal, chemical, and mechanical properties, ease of synthesis, reusability, low cost, and high absorption capacity are only a few of their many benefits [93]. Recently, the application of SPE with IIPs (IIP–SPE) for elemental speciation analysis has attracted extensive research interest.

**Figure 2:**
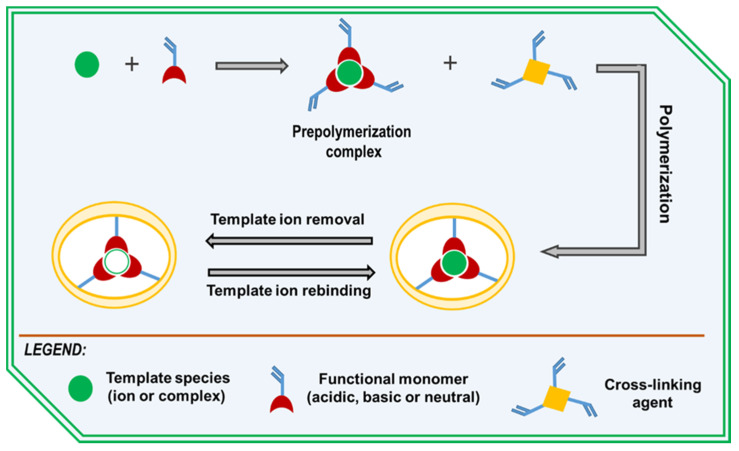
Scheme of the IIPs preparation process.

The synthesis of IIPs involves several main steps (Figure 2) [93,94,95]. Initially, a prepolymerization complex is formed by noncovalent interactions (chelation, electrostatic interactions and hydrophobic interactions) between functional monomers and a template (ions or a complex between ions and a chelating agent). In the second step, this complex is copolymerized with a cross-linking agent is carried out, which leads to the stabilization of the binding cavities in a highly cross-linked three-dimensional network copolymer. Finally, the template ion is removed from the copolymer matrix using suitable extractants, and thereby, creating specific cavities with a size, shape and chemical functionality complementary to those of the template species.

The methods used to obtain the IIPs have been described in detail in several reviews [96,97,98]. Briefly, these are free radical polymerization (bulk, precipitation, suspension, and emulsion), graft polymerization, reversible addition-chain fragmentation (RAFT) polymerization, so–gel processes, etc. The main components used in the synthesis of IIPs are the functional monomers (acidic, basic or neutral), cross-linkers, template species, solvents (porogens), and initiators. The correct choice of these components is of key importance because it affects both the stability of the formed complex before and during the polymerization process and determines the subsequent ability of the IIP to interact selectively with the target ion.

Depending on the method of incorporation of the metal ions into the polymer matrix, the following four main approaches for the synthesis of IIP are known: (1) cross-linking of linear chain polymers carrying metal-binding groups with a bifunctional reagent, (2) chemical immobilization of complexes of metal ions with vinyl groups containing ligands, (3) trapping of the nonvinylated chelating ligand in the pores of the polymer matrices and (4) surface imprinting in which binding sites are mainly formed at or near the core surface [92,96,98,99].

#### 2.7.1. IIPs Prepared by Chemical Immobilization

The “chemical immobilization” approach is also called direct imprinting because the metal ions initially form complexes with ligands containing polymerizable vinyl groups. The prepared prepolymerization complexes are then copolymerized with a cross-linking monomer and form a three-dimensional polymer network. Although the application of this approach is simple, it has been observed that high selectivity is not achieved when commercial monomers (MAA, 4-VP, 1-vinylimidazole (1-VIA), acrylamide (AA), hydroxyethyl methacrylate (HEMA), etc.) are used [96]. For this reason, the synthesis of novel functional monomers containing more complex and selective ligands is required.

As(III)-IIP has been prepared by cross-linking copolymerization of 1-VIA as a functional monomer, DVB as a cross-linker, in the presence of As(III) ions [100,101]. The strategy used to determine the inorganic arsenic (iAs) species by SPE with this IIP is “Retention of two or more species is followed by their simultaneous elution and subsequent chromatographic separation and determination” [83]. The developed IIP-SPE procedure provides the quantification of very low concentrations (lower than 0.02 mg/kg for As(V)) of these two toxic As species in commercially available fish [100]. The high selectivity of the material offers the possibility of iAs speciation after HPLC separation. The absence of retention of organoarsenic compounds (mainly arsenobetaine) on the sorbent suggests that coelution was avoided, allowing for the precise measurement of low concentrations of As(III) and As(V). The same IIP is also used to develop the vortex-assisted dispersive micro-SPE protocol for rapid isolation and preconcentration of iAs species in extracts from rice samples prior to their determination by HPLC coupled to ICP-MS [101].

Li et al. developed an analytical procedure for the determination of organic arsenic compounds (oAs) in feeds, edible chicken and pork samples based on SPE with molecular imprinted polymer (MIP-SPE) coupled with HLPC [102]. A new functional monomer (N,O-bismethacryl ethanolamine (NOBE)) was synthesized and used. The imprinted adsorbent was successfully prepared by the suspension polymerization with the initiator and RAFT agent and used for four phenylarsonic compounds (arsanilic acid (ASA), roxasone (ROX), nitarsone (NPA) and carbarsone (CBA)). A good recognition performance was achieved with a high volumetric adsorption capacity, robust selectivity and rapid mass transfer process. Compared with the traditional SPE column, this MIP-SPE column displayed a higher recovery and better cleanup performance owing to its high selectivity. The recoveries were calculated as 83.4% to 95.1% with the different samples.

Molecular imprinting technology has been employed to prepare a specific affinity chromatographic stationary phase for speciation of organotin species [103]. Synthesis of polymer beads with satisfactory shape and size for chromatographic purposes was achieved using different polymerization methods. Three different types of materials were obtained: (i) a composite material, (ii) a polymer prepared via-Iniferter grafting; (iii) an emulsion polymer. Tributyltin (TBT) was used as the template molecule and the noncovalent approach was applied. The organotin species were separated and determined by LC-ICP-MS. Satisfactory resolution of all four OTCs studied (dibutyltin (DBT), monobutyltin (MBT), tributyltin (TBT) and triphenyltin (TPhT)), was achieved in 20 min using the emulsion polymer. The detection limits achieved were similar to those obtained with commercial stationary phases (6 pg for MBT; 10 pg for both TBT and TPhT; and 20 pg for DBT). The elimination of matrix interferences and the achievement of good recovery for all species, including MBT, are the primary benefits of the suggested stationary phase based on IIP. The method proposed was validated using two biota reference materials (ERM-CE477 mussel and T-38 oyster).

A novel method based on As(V)-IIP supported on a thin layer chromatography (TLC) plate was developed and applied to the separation of inorganic arsenic species in aqueous media [104,105]. A new nanostructured As(V)-IIP was synthesized by ultrasound-assisted precipitation copolymerization of itaconic acid (ITA) and ethylene glycol dimethacrylate (EGDMA) [104]. The prepared As(V)-IIP was used to fabricate a TLC plate on which the separation of As(V) from As(III) was possible. The separated zones were transferred to an ICP-MS instrument utilizing a laser ablation technique (LA-ICP-MS). It was found that the type and pH of the mobile phase can critically affect the successful separation of the two iAs species on the IIP-based TLC plate. A similar analytical procedure based on Sn(II)-IIP was proposed for the speciation of inorganic tin species in water and plasma samples [105]. For this aim, nanostructured Sn(II)-IIP was synthesized by precipitation polymerization using N-allylthiourea (NATU) and EGDMA in the presence of Sn(II) ions. TLC plate prepared with this material was capable of separating Sn(II) and Sn(IV) ions. After completion of the separation process, the plate surface was scanned by LA-ICP-MS. The developed method only applies to samples with a pH between 4 and 7 and is not applicable to solid samples that require acid digestion.

#### 2.7.2. IIPs Prepared by Trapping Technique

The trapping approach is the most commonly used method to IIPs preparation. The synthesis scheme includes one additional step compared to direct imprinting—the formation of a complex between template ion and specific chelating agent, which is then incorporated into the polymer network. The high selectivity of IIPs can be explained by the polymer memory effect toward the metal ion interaction with a specific ligand, coordination geometry, metal ion coordination number, charge and size. For this reason, the choice of chelating agent is of key importance.

An SPE method using Sb(III)-IIP as a sorbent combined with ETAAS was applied to determine antimony species in water samples and total antimony in fruit juice [106]. The Sb(III)-IIP has been synthesized by precipitation copolymerization of ST and EGDMA in the presence of Sb(III)–APDC complex as a template. It was found that Sb(III) ions were quantitatively retained on the sorbent at pH 5. Total antimony was determined after the reduction of Sb(V) to Sb(III). The procedure is simple and reproducible. The main benefits of the proposed method are the rejection of the matrix constituent in ETAAS determination, the enhancement of the sensitivity, low cost, and high enrichment factor. Yordanova et al. developed nonchromatographic method for the determination of Sb(III) based on selective SPE of the analyte with a novel Sb(III)-IIP as a sorbent followed by ICP-OES measurement [107]. In the preparation process of the IIP, 2-mercapto-N-(2-naphthyl)acetamide (thionalide) was used as a new chelating ligand, resulting in an excellent extraction efficiency and selectivity toward Sb(III). The major advantages of the method developed are easy operation, fastness and low reagents consumption with no need of any additional sample pretreatment. The reported procedure was applied to natural water samples, demonstrating very good accuracy and reproducibility, enabling 25-fold preconcentration of target chemical species Sb(III).

A trapping method was used to synthesize several Cr(III)-IIPs, which were incorporated into Cr speciation schemes [108,109,110,111]. The authors used various chelating reagents (Cr(III)–PDC complex, Cr(III)-8–HQ complex, Cr(III)–nicotinate complex and Cr(III)-1,10–phenanthroline complex) as template species. The synthesized Cr(III)-IIPs showed higher selectivity towards Cr(III) than to Cr(VI). Trzonkowska et al. show that Cr(III)-IIP prepared by imprinting the Cr(III)-1,10-phenanthroline complex in the presence of two functional monomers (ST and 4-VP) leads to stronger binding of this complex in polymer matrix and improved selectivity of the sorbent towards Cr(III) ions in the presence of Cu(II), Mn(II) and Fe(III) ions, compared to the imprinted polymer containing only ST [111]. Multicommutation flow system-FAAS system was also developed, which allows automation of the separation procedure of Cr(III) ions on Cr(III)-IIP, reduces the required volume of sample and eluent, shortens the analysis time and improves the repeatability of the separation process (RSD < 3%) [109].

Mitreva et al. reported a procedure for the determination of Fe(III) and total Fe(II) + Fe(III) in wine samples employing newly synthesized Fe(II)IIP as a sorbent incorporated in dispersive SPE [112]. Fe(II)-IIP was prepared by employing trapping technique using Fe(II)−2,2′-bipyridyl (Fe(II)-BP) complex as a template. The experiments performed showed the high selectivity of the sorbent toward Cd(II), Cu(II), Mn(II), Pb(II) and Zn(II) but low selectivity toward Fe(III). It might be concluded that ion-imprinted polymers could not be used for the selective separation of metal ions with only different oxidation states if template complexes had similar stoichiometry. However, the separation of both Fe species was achieved in the presence of fluoride ions as a masking agent for Fe(III). The same group also investigated the effect of template species (Fe(II) complexes with 4-(2-pyridylazo)resorcinol (PAR) or BP) as well as the functional monomers (MAA or HEMA) on the extraction efficiency and applicability of Fe(II)-IIPs for iron speciation in surface water [113]. All the results indicate that Fe(II)-IIP synthesized with MAA as a functional monomer and Fe(II)–PAR complexes as template species showed the best binding properties and higher selectivity toward Fe(II).

Roushani et al. [114] and Ara et al. [115] synthesized Fe(III)-IIP using Fe(III)-3,6-bis (3,5-dimethyl-1-H-pyrzol-1-yl)-1,2-dihydro–1,2,4,5-tetrazine complex and Fe(III)–HQ complex as template species, respectively. In these papers, distribution coefficients (*K*_d_) are shown for synthesized IIPs for Fe(III) in the presence of Fe(II) ions, but the analytical methods developed are still applied solely for Fe(III) determination.

Mercury speciation based on IIP-SPE is usually performed by Approach 1 or 2. IIPs for Hg(II) has been prepared by cross-linking copolymerization of MAA and trimethylolpropane trimethacrylate (TMPTMA) in the presence of Hg complexes with two chelating agents: with 1-(2-thiazolylazo)-2-naphthol (TAN—specific chelating reagent for mercury) (P(TAN-Hg)) [116] or with 1-pyrrolidinedithiocarboxylic acid (PDC—nonspecific reagent, forming complexes with various metals ions) (P(PDC-Hg)) [117]. The Hg(II)-IIPs synthesized demonstrated good selectivity toward template species Hg(II), which could be explained again with the assumption that configurations of ligands exhibit maximum activity for Hg(II) complex formation because the coordination geometry is completely the same as in the complex. This hypothesis is confirmed by the low value of the capacity of the control polymer containing only TAN molecules incorporated into the polymer network. In this case, the TAN molecules are blocked for the formation of the Hg(II)–TAN complex at SPE. The optimal pH value for the quantitative sorption is 7. The adsorption capacity of P(PDC-Hg) was found to be greater than that of P(TAN-Hg). The P(TAN-Hg) and P(PDC-Hg) were applied for the speciation of Hg in surface water: Hg(II) was determined selectively in nondigested sample, while total mercury (sum of inorganic and methylHg) was determined in the digested sample. Dakova et al. also presented a critical comparison between the extraction efficiency, adsorption capacity and selectivity of Hg(II)-imprinted copolymer gels prepared with VIA (basic), MAA (acidic) or HEMA (neutral) as functional monomers [118]. Hg(II)-IIPs were synthesized by dispersion copolymerization of the functional monomer, TMPTMA as cross-linking agent and AIBN as an initiator in the presence of Hg(II)–TAN complexes. All prepared Hg(II)-IIPs have excellent selectivity towards Hg(II) over methylHg. They are characterized by fast sorption/desorption kinetics and good chemical and mechanical stability. Better binding properties and the highest selectivity toward Hg(II) were observed for imprinted copolymer gel based on VIA as a monomer. The application of this Hg(II)-IIP for the determination of inorganic and methylmercury in surface water samples was demonstrated.

A novel methylHg-IIP, which contains methylHg complex with phenobarbital as a template, has been synthesized by precipitation polymerization technique, and it has been used in analytical procedure for Hg speciation by HPLC-ICP-MS. [119]. Trace levels of Hg(II), methylHg, and ethylHg have been retained on a column-packed with prepared methylHg-IIP at pH 8.0 and at a flow rate of 2.0 mL/min. The retained Hg species were eluted, separated on a Kinetex C18 column working under isocratic conditions and quantified by ICP-MS. The developed method was successfully applied for Hg speciation in several seawater samples from unpolluted areas. The same methylHg-IIP was used to assess low concentrations of Hg(II) and methylHg in edible seaweeds by LC-ICP-MS measurements after column preconcentration [120]. Mercury species were first isolated from seaweed by ultrasound-assisted extraction using a 0.1% (*v*/*v*) HCl, 0.12% (*w*/*v*) L-cysteine, 0.1% (*v*/*v*) mercaptoethanol solution.

A new IIP for Sn(IV) has been synthesized and applied for selective SPE of inorganic tin species [121]. The precipitation polymerization was performed using MAA (functional monomer), EGDMA (cross-linking agent) and a complex between Sn(IV) and PAR as a template species. The concentration of Sn(II) was calculated by subtracting Sn(IV) from total Sn. ETAAS was used as an instrumental method for selective determination of Sn(IV) and Sn(II) in food and water samples.

#### 2.7.3. IIPs Prepared by Surface Imprinting

The IIPs prepared with conventional methods have some disadvantages, such as incomplete template removal, small binding capacity and slow mass transfer due to the difficulty of the ions accessing the specific sites incorporated within the highly cross-linked polymer network. These problems can be solved by using the surface imprinting approach. IIPs prepared by this technique show many advantages, including high selectivity, more accessible sites, faster mass transfer and binding kinetics, and easier elution because the imprinted cavities are located exclusively in the surface shell of the particles [122].

The ideal support of core–shell materials could be silica gel particles which have mechanical/chemical stability, low cost, and ease of preparation and functionalization. Chromate anion-imprinted sorbents supported on core (silica gel or micrometer-sized silica spheres) for nonchromatographic Cr speciation in surface water and textiles have been developed by Mitreva et al. [123] and Vasileva et al. [124]. The preparation procedure is based on the grafting of 3-methyl-1-trimethoxysilylpropylimidazolium, preliminarily coordinated to CrO_4_^2−^ as a template ion, onto the surface of the core. An excellent separation of Cr(VI), selectively retained on the sorbent, from Cr(III), which remained in the solution, was achieved at pH 2–3 after 20 min. A freshly prepared mixture of ascorbic acid and nitric acid was selected as the most efficient eluent for quantitative desorption of the retained Cr(VI).

New Hg(II) ion-imprinted polymers layer-coated silica gel particles (Hg(II)-IIP) were synthesized and applied for the determination and speciation of Hg in wine samples [125]. Recovery experiments performed for selective determination of Hg(II) in wines showed that the interfering organic matrix did not influence the extraction efficiency when Hg(II)–PDC complex was used as a template in the copolymer matrix. The analytical scheme proposed consists of two steps: (1) selective determination of Hg(II) in a nondigested wine sample; (2) determination of total Hg (the sum of Hg(II) and methylHg) in a digested wine sample. The content of methylmecury is defined as the difference between these two measurement results. In comparison with other known methods for mercury determination and speciation in wine samples, the proposed method is cheaper and faster and provides comparable LODs without derivatization steps and hyphenations with GC.

Liu et al. used APTES as a silane coupling agent, and a Cr(III)-IIP was prepared by an easy one-step sol–gel reaction with a surface imprinting technique on the support of mesoporous silica material [126]. Under the optimal conditions, Cr(III) was absorbed quantitatively, but Cr(VI) was not retained. A novel Cr(VI)-IIP was synthesized on the surface of mesoporous silicon (SBA-15) using MAA and 4-VP as bifunctional monomers and Cr(VI) anion as template ion by surface thermal initiation imprinting technique [127]. Cr(VI)-IIP prepared possesses a strong selective adsorption ability for Cr(VI) in the presence of competing ions such as Cr(III), Cu(II), Cd(II) and Ni(II), which demonstrated that Cr(VI)-IIP could be applied in complex aqueous environments to achieve efficient separation of Cr(VI) while enabling the separation of different valence states of elemental Cr.

Zhang et al. synthesized sorbent based on novel IIP for Hg(II) by a sol–gel process in the presence of dithizone–Hg(II) chelate complex [128]. The resultant Hg-IIP offered high binding capacity and fast kinetics. The optimum pH range for the quantitative adsorption of Hg(II) from aqueous solutions is 7.0–8.0. Hg speciation analysis was carried out by using IIP-SPE coupled with AFS. Its applicability was demonstrated for the analysis of Hg(II) in seawater and lake water samples, together with organic mercury species in human hair and fish meat samples. The method developed demonstrated significant application perspectives for rapid enrichment, highly effective cleanup and determination of trace Hg species in complicated matrices.

In recent years, magnetic IIPs have proven to be extremely valuable and useful sorbents for SPE due to their magnetic sensitivity. Türkmen et al. fabricated IIP-based magnetic nanoparticles materials for As(III) and As(V) removal from wastewater [129]. As(III)- and As(V)-IIPs were synthesized using a molecular imprinting technique in the presence of iron oxide (Fe_3_O_4_). N-methacryloyl-(l)-cysteine (MAC) was used as a functional monomer. IIP nanoparticles displayed considerable removal ability for As(III) and As(V) in a wide pH range of 4–8. The IIPs were selective for arsenic toward other analogs such as NO_3_^−^, PO_4_^3−^, SO_4_^2−^, AsO_2_^−^, HAsO_4_^−^. The kinetic and isotherm studies show that the adsorption process was monolayer rate-limiting kinetics on the homogeneous polymer surface. New core–shell magnetic MIPs (MMIP) were prepared and applied for selective adsorption and special recognition of ROX from water [130]. In this study, on a core of Fe_3_O_4_@SiO_2_ nanocomposite, a shell of ROX-MIP, containing copolymer of 2-VP and EGDMA, was chemically bonded. The sorption efficiency of MMIP to ROX was shown to remain unchanged over a wide pH range (3.0–10.0). The *K*_d_ value of MMIPs for ROX, *p*-ASA and As(V) were 17.03, 3.66 and 1.39 L/g, confirming the specific binding of MMIPs to ROX in water.

Magnetic Cr(VI)-imprinted nanoparticles (Fe_3_O_4_@Cr(VI)-IIPs) were prepared by hyphenating surface ion-imprinted with sol–gel techniques [131]. During the preparation process, 1-VIA and APTES were used as organic functional monomer and comonomer. Methacryloxypropyltrimethoxysilane (γ-MAPS) was selected as the coupling agent to form the covalent bonding between organic and inorganic phases. A facile, rapid and selective dispersive SPE method for the extraction and enrichment of Cr(VI) prior to FAAS was developed.

A new methylHg ion-imprinted magnetic nanoparticle (methylHg-IIMN) for specific separation and preconcentration of ultratrace methylHg from an aqueous sample was prepared by Jiang et al. [132]. The methylHg-IIMN has been synthesized on the surface of Fe_3_O_4_@SiO-γ-MAPS NPs via thermal polymerization of MAA (functional monomer), TMPTMA (cross-linker), and AIBN (initiator) in the presence of methylHg–PDC complex (template). It has been established that this sorbent can specifically recognize and extract methylHg (pH 5) in the presence of other organic Hg species. Combined with CE-ICP-MS, methylHg-IIMN can be used for the simple and accurate detection of ultratrace methylmercury in natural water within 50 min with a recovery of 92–99%, an RSD < 8% n = 5) and a LOD of 0.084 pg/mL.

An interesting and promising application of IIPs is their use in diffusive gradients in thin films (DGT) technique. This technique was proposed by Davison and Zhang [133] and is a convenient speciation tool. It has a number of advantages such as simplicity, low cost, no power requirement, selective accumulation of labile analyte’s species, in situ preconcentration, minimization of chemical interference and ability to obtain accurate results. DGT technique has been successfully used to determine the kinetically labile fraction of metals, which is the sum of free ions, inorganic and weak organic complexes in water, or to assess the bioavailability of metals. For the first time, Fan et al. developed a DGT device for the analysis of free Cd(II) species, based on Cd(II) ion-imprinted sorbent (IIS) as the binding agents and commercial polyethersulfone membrane (PES) as diffusion layer (PES/IIS-DGT) [134]. This device layer can accurately measure free Cd(II) species at environmentally relevant levels. The PES/IIS-DGT provides reliable results over a large ionic strength (0.001–0.7 mol/L) and pH range (4.5–7.5). The measurement of free Cd(II) species in a synthetic solution containing different concentrations of ligands by PES/IIS-DGT showed excellent agreement with the value measured by Cd(II) ion selective electrodes. A new DGT device using Pb(II) ion-imprinted silica (IIS) as binding agents and a commercial cellulose acetate dialysis (CAD) membrane as a diffusion layer (CAD/IIS-DGT) was also constructed, which was successfully applied to measure free Pb(II) species in synthetic solutions, in natural fresh water and in industrial wastewater [135].

Table 3 presents an overview of the reported synthesis of IIPs and analytical procedures proposed for the separation and speciation of chemical elements using IIPs.

### 2.8. Polymeric Monoliths as Sorbents for Solid-Phase Extraction

Organic–inorganic hybrid monolithic columns prepared via sol–gel process combine the advantages of traditional organic polymer and inorganic silica monolithic columns with good biocompatibility, large specific surface area and high mechanical stability. The preparation method to directly introduce functional groups into the skeleton of monolith during sol–gel process, called “one-pot”, can make the monolith more uniform and reproducible than the postmodified ones. A thiol- and amine-bifunctionalized organic–inorganic hybrid monolithic column was prepared via one-pot cocondensation of 3-mercaptopropyltrimethoxysilane, N-(2-aminoethyl)-3-aminopropyltriethoxysilane and tetraethylorthosilicate, and applied for separation and enrichment of inorganic arsenic species [136]. Both As species were retained simultaneously and further separated by selective eluents. Using the same approach, a novel carboxyl-functionalized hybrid monolithic column was developed based on “thiol–ene” click reaction via “one-pot” by choosing mercaptosuccinic acid, γ-methyl methacrylate trimethoxysilane and tetramethoxysilane as reaction monomers [137]. The hybrid monolith column selectively retained Cr(III) while Cr(VI) passed through the column. Both species were measured in the same sample after Cr(III) elution using ICP-MS.

Speciation of mercury is still an analytical challenge from both points of view—very low natural environmental concentration, instability of chemical species usually prone to transformations during the extraction and separation step and complex retention behavior of different Hg species. The application of a polymer monolithic column prepared by radical polymerization is a kind of answer to these problems—functionalization with specific groups ensures fast and reliable selectivity to interest elements and their species.

It might be expected that for the speciation of Hg the introduction of mercapto groups will ensure the best efficiency of enrichment and separation due to the strong affinity between them and Hg species. In addition, abundant functional sites, as well as the porous structure of polymeric monoliths, would be expected to improve selectivity and extraction kinetics. Successful Hg enrichment and speciation were demonstrated by using mercapto functionalized capillary monolithic column [138]. The synthesis was based on a one-step strategy, which involves simultaneous radical polymerization and initiation of thio–lene click reaction. The realized system chip-based array monolithic capillary microextraction consists of the described column embedded in the microchannel of the microfluidic chip and was applied for Hg mercury speciation in cells. The detection system combines both ICP-MS and HPLC ICP-MS detection and ensures reliable determination of iHg and methylHg in HepG2 cells.

Novel specific toward Hg species monolith doped with Fe_3_O_4_ (TSM@Fe_3_O_4_) was in situ prepared in a capillary using vinyboronic anhydride pyridine complex and styrene as dual monomers, and EGDMA and DVB as mixed cross-linkers [139]. The TSM@Fe_3_O_4_ was used as a sorbent of magnetic field-reinforced in-tube solid-phase microextraction (MFR/IT-SPME) for efficient extraction of Hg species under the assistance of a magnetic field. Four mercury species, including methylHg, ethylHg, phenylHg and Hg(II), were first coordinated with dithizone to form chelates. After the optimization of extraction parameters, the introduced MFR/IT-SPME was online hyphenated with HPLC/DAD and successfully applied to determine trace Hg species in environmental water. Limits of detection varied from 0.0067 to 0.016 μg/L, and the RSDs were below 7.5%.

Capillary microextraction (CME) is a highly efficient pretreatment technique characterized by fast mass transfer and low reagent consumption. Its efficiency depends on the capillary column used. In recent years, monolithic capillaries received serious attention due to their high extraction capacity and relatively easy preparation. Capillary microextraction was applied for Se speciation using polymer monolithic capillary based on glycidyl methacrylate as a monomer and EGDMA as cross-linker, functionalized with ethylenediamine [140]. The capillary column was incorporated in a flow injection system online coupled to ICP-MS. The selective retention of Se(IV) was found at the pH range of 2–2.8, while sorption of Se(VI) is negligible. Total Se was determined after Se(IV) oxidation. The speciation procedures were applied for natural water. A new mercapto functionalized polymer monolithic capillary was prepared after modification of poly(glycidyl methacrylate-ethylene dimethacrylate) monolithic capillary with cystamine and used for the selective adsorption of Sb(III) and Te(IV) based on the affinity difference of -SH group toward the inorganic species of Sb and Te [141]. An online strategy of CME-ICP-MS was developed for the simultaneous determination of inorganic Sb species and inorganic Te species in water samples. The method possesses the merits of simple sorbent preparation, strong resistance to matrix interferences, low consumption of organic solvent, and low detection limits—3.9 and 5.9 ng/L for Sb(III) and Te(IV), respectively. The accuracy of the method was verified by CRM of Environmental Water (GSB07–1376-2001, GBW(E)080548).

### 2.9. Metal–Organic Frameworks

**Figure 3:**
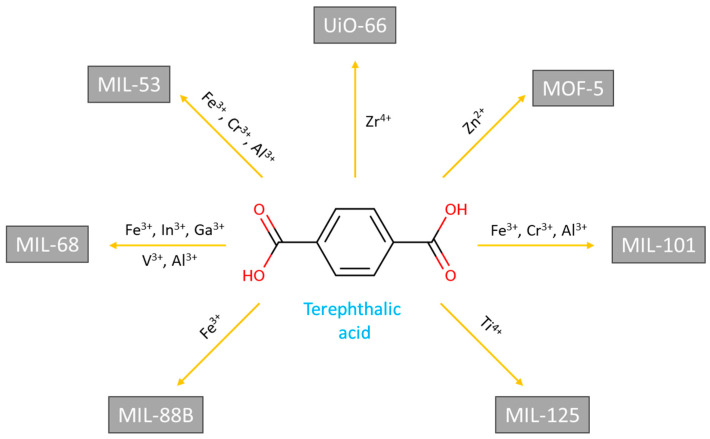
Metal–organic frameworks based on terephthalic acid as a linker.

Metal–organic frameworks (MOFs) are coordination polymers consisting of metal ions or clusters coordinated to organic ligands to form extended three-dimensional structures. Due to their properties, such as high surface area, tunable pore size, and mechanical and thermal stability, they have gained significant attention for their applications in various fields, including adsorption-based separation processes [142,143,144,145]. The coordination of metal ions and organic ligands leads to the formation of porous frameworks with structures that are strongly dependent on the particular metal ion and specific ligand, therefore, the size and shape of these pores can be tuned by choosing specific ligands towards particular metal ions. Coordination polymers exhibit a high degree of structural diversity due to the wide range of available metal ions and ligands (Figure 3). This versatility allows for the design of materials with specific properties tailored for various applications. Up to now, MOFs still find limited applications in the field of SPE-based speciation analysis.

MIL-101 is a well-known group of MOFs typically composed of benzene 1,4-dicarboxylic (terephthalic) acid and Cr, Fe, or Al as metal nodes [143]. In the common case, this type of MOF has been involved in the preparation of magnetic composites for selective SPE of Cr(VI) [146], Se(IV) [147], Sn(IV) [148], V(V) [149], and Tl(I) [150], following an identical conception. Magnetite nanoparticles were initially mixed with tetraethyl orthosilicate (TEOS), resulting in the synthesis of Fe_3_O_4_@SiO_2_ NPs, and subsequently modified with an appropriate ligand, such as 2-(propylamino-ethyl) dithiocarbamate [146], amino dithiocarbamate [147], 1-(2-pyridylazo)-2-naphthol [148], morin [149], and 4-aminodibenzo-18-crown-6 [150]. Finally, the composite sorbents were prepared via immobilization of the modified Fe_3_O_4_@SiO_2_ NPs in the structure of metal–organic frameworks MIL-101(Fe) [146,149] or MIL-101(Cr) [147,148,150]. Speciation procedures were applied to water samples in accordance with the A1 scheme (Figure 1) and combined with ETAAS measurements for the analytes quantitation [147,148,149,150], with the exception of Cr speciation, where Cr(VI) was extracted at pH 2, while SPE of total Cr was conducted at pH 5 without any need for additional treatment for the oxidation of Cr(III) to Cr(VI) [146]. As regards the sorption mechanism, retention of the target species relied on complexation with the ligands grafted onto Fe_3_O_4_@SiO_2_ NPs, while the role of MOFs was mentioned as a support with a high surface area and spacer for prevention of NPs aggregation [146,148,149,150]. On the other side, comparisons between the sorption capabilities of bare MIL-101 functionalized and bare Fe_3_O_4_@SiO_2_ NPs, and the composites undoubtedly showed superior extraction efficiencies for the last ones, which is probably due to some kinds of synergistic effects, i.e., electrostatic attraction between positively charged species and negatively charged sites of MILs-101 [148,149]. A similar approach for speciation analysis of inorganic arsenic was formerly developed by using a nanocomposite consisting of ditiocarbamate-modified Fe_3_O_4_@SiO_2_ NPs and copper-based MOF-199 (benzene-1,3,5-tricarboxylate as an organic linker) [151]. Another implementation of MIL-101(Fe) in arsenic speciation was reported by Aslan and Tor [152]. After synthesis of the MOF, it was suspended in acetone and then mixed with poly(vinylidene fluoride) to produce a miniaturized MIL-101(Fe) mixed-matrix membrane (MOF-MMM). The selectivity of MOF-MMM towards As(V) at pH 3–6 was explained by the positively charged MIL-101(Fe) at pH < 6 resulting in the electrostatic attraction of the anionic H_2_AsO_4_^−^ and HAsO_4_^2−^ while As(III) remained in the solution as the neutral molecule H_3_AsO_3_. Separation of the inorganic As species was carried out at pH 6, followed by direct determination of As(V) on the MOF-MMM by EDXRF measurement, avoiding the elution step. This great benefit can be considered a counterpoint to the drawback of a long equilibrium time (at least 120 min) [152].

UiO-66 is the first synthesized MOF from the group UiO, named after the University of Oslo [153]. Composed of Zr-clusters as metal nodes and terephthalic acid as a linker, UiO-66 possesses a highly porous structure with a large surface area and exceptional stability. Volynkin et al. developed a procedure for mercury speciation analysis using UiO-66 in two different modes (nonmodified and cysteine-modified), aiming for the determination of Hg^2+^, phenylHg^+^, and methylHg^+^ in water samples [154]. It was experimentally verified that nonmodified UiO-66 was able to sorb only organic Hg chemical forms, while all studied species were captured by the cysteine-modified Zr–MOF, and thus, the concentration of inorganic Hg^2+^ was found as a difference. Quantitations were realized directly from the solid-phase using thermal release-electrothermal atomic absorption spectrometry (TR-ETAAS) with no need for elution. In arsenic speciation analysis, a magnetic Zr–terephtalate MOF composite was used for online column SPE of As(V), MMAs and DMAs followed by sequential elution with ammonia solutions with different concentrations and ICP-MS determination of each studied As species [155]. A second column packed with dimercaptosuccinic acid-modified magnetic Zr–MOF composite was involved in the developed online system to ensure the retention of As(III) at pH 6 based on the strong affinity between -SH (soft base) and As(III) (soft acid). Desorption of As(III) was achieved with a 2% solution of thiourea in 0.5 M HNO_3_, and thus, the four investigated As species were successfully quantified in spiked squamous carcinoma cell samples [155].

Another magnetic UiO-66 composite was synthesized using 2,5-dimercaptoterephthalic acid and ZrCl_4_ to prepare a sulfur-functionalized sorbent (Fe_3_O_4_@UiO66-SH) for simultaneous SPE of Hg^2+^ and CH_3_Hg^+^ combined with HPLC-ICP-MS system for the separation and quantitation of individual Hg species in water and fish samples [156]. Desorption of both Hg^2+^ and CH_3_Hg^+^ from Fe_3_O_4_@UiO66-SH was performed with 1 mL nitric acid solution (0.5%, *v*/*v*) containing thiourea (2%, *m*/*v*), and thus enrichment factors 45.7 and 47.6 were accomplished, respectively. Applications of MOFs in SPE-based methods for speciation analysis are summarized in Table 4.

### 2.10. Covalent Organic Frameworks

Covalent organic frameworks (COFs) are porous, crystalline materials formed through covalent bonds between organic molecules. They have a well-defined structure of extended two- or three-dimensional networks with repeating structural units, and similarly to MOFs, COFs have a large surface area. Furthermore, COFs have been proven to possess higher resistance in water and acidic solutions compared with MOFs, which may be considered a great advantage for working in a broader range of chemical conditions and facilitating their implementation in the SPE of trace elements [157].

The utilization of COFs in the field of speciation analysis is still sparsely represented. Fabrication and application of COF-based electrospun composite nanofiber membranes were reported by Kang et al. [158]. The sorbent material was synthesized via a reaction between 1,3,5-trialdehyde phloroglucinol (Tp) and 3,3′-dinitrobenzidine (DNB), followed by treatment with SnCl_2_ to produce the COF TpBD(NH2)2. As a second step, the electrospinning technique was applied to prepare nanofibers by adding polyacrylonitrile (PAN) to the dispersed TpBD(NH_2_)_2_. As-synthesized composite membrane PAN@TpBD(NH_2_)_2_ was involved in a method for the determination of inorganic As in rice based on pipette-tip solid-phase extraction and HG-AFS measurement. Acting as a strong anion exchanger, the sorbent was able to capture the anionic species of As(V) at pH 6 due to the protonation of amino groups, while the sorption of As(III) was around 40% under the same conditions. On the other side, As(III) could be easily oxidized to As(V) during the microwave-assisted acid digestion (HNO_3_-H_2_O_2_) of real rice samples; therefore, the method was proposed for the determination of inorganic As.

Thiol and thioether-functionalized magnetic covalent organic frameworks were successfully implemented in the speciation analysis of mercury, demonstrating a strong affinity toward inorganic Hg, methylHg, and ethylHg [159]. The urchinlike sorbent (Fe_3_O_4_@COF-S-SH) was synthesized by coating Fe_3_O_4_ nanoparticles with COF (2,5-divinylterephthalaldehyde and 1,3,5-tris(4-aminophenyl) benzene), followed by grafting of 1,2-ethanedithiol. Simultaneous adsorption of iHg, methylHg, and ethylHg was accomplished at pH 2–9, achieving enrichment factors of 370, 395, and 365, respectively. Separation and quantitation of the studied Hg species were realized with HPLC-ICP-MS, and the proposed method was applied to Hg speciation in water and fish samples.

### 2.11. Biopolymers

Among the large diversity of sorbent materials for the different solid-phase extraction techniques, biosorbents still attract enormous attention because of their numerous advantages, such as low toxicity, biodegradability, biocompatibility, and chemical functionality. Polysaccharides and polypeptides are typical representatives of polymeric materials with a natural origin, also known as biopolymers, possessing good adsorptive capabilities owing to the large number of different functional groups. Biopolymers, as well as biopolymeric derivatives and composites, are widely used for water treatment and removal of environmental pollutants, dyes, pesticides, heavy metals [160,161,162,163]. Natural polymeric materials, such as chitosan [164,165,166,167,168,169,170], cellulose [171,172,173,174,175,176,177,178], β-cyclodextrin [179,180], dextran [181], and peptides [182], have also found many applications in elemental speciation analysis of chromium [164,165,166,169,173,175,176,177,180,181,182], arsenic [167,174,178], mercury [171], selenium [168,172,174], manganese [179], and silver [170]. In the common case, biopolymers have been chemically modified or involved in the preparation of composite materials in order to improve extraction efficiency toward the target chemical species of the elements.

#### 2.11.1. Chitosan-Based Materials

Chitosan is a linear polysaccharide derived from poly-N-acetyl-D-glucosamine (chitin) by partial deacetylation, therefore, it is a natural heteropolymer composed of D-glucosamine and N-acetyl-D-glucosamine units. The higher degree of chitin deacetylation results in the preparation of chitosan with a large number of amino groups, which can be easily protonated in an acid environment and thus electrostatically attract the negatively charged chemical species, e.g., arsenates and selenates. Such a mechanism was proposed by Boyaci et al. for the selective separation of As(V) from As(III) in water samples by using chitosan and chitosan-immobilized sodium silicate [167]. At a low pH value (3.0), H_2_AsO_4_^−^ was quantitatively sorbed by both of the reported materials, while the degree of sorption for As(III) (under the same experimental conditions) was below 15%. Moreover, the authors also evaluated the adsorptive effectiveness of chitin for the retention of inorganic arsenic species and clearly showed that it was not applicable for the speciation analysis of As because the sorption of both As(V) and As(III) did not exceed 10%. In addition, the immobilization of chitosan onto a supporting surface increases the surface area of the sorbent (compared to chitosan itself), with a corresponding increase in the number of functional groups available for sorption [167]. From a practical viewpoint, the usage of chitosan as an adsorbent in its pure form has some limitations, such as the relatively high solubility in weakly acidic solutions [183,184]. A good way to overcome this drawback is the cross-linking of chitosan with appropriate bi- or polyfunctional reagents, e.g., glutaraldehyde [175,183], diethylene triamine [168], and ethylene glycol diglycidyl ether [169]. In general, cross-linking is a convenient approach to extend the chemical resistance and mechanical strength of chitosan-based sorbents. In some cases, the binding ability of such materials was reported to be lower than that of pure chitosan, but this was not considered an obstacle if the concentration of the analytes was low [183]. Cross-linked chitosan was successfully implemented in the selective separation and preconcentration of selenate in environmental water [168]. The predominant forms of Se(VI) at pH 3.6 (SeO_4_^2−^ and HSeO_4_^−^) were strongly attracted by the positively charged amino groups of the sorbent, while Se(IV) remained in the solution as a neutral molecule. Retention of both Se(VI) and Se(IV) on the cross-linked chitosan at pH 3.6 was 94% and 5%, respectively. The choice of diethylene triamine as a cross-linking agent ensured not only the chemical resistance of the sorbent in acidic solutions but also enhanced its ability to sorb the anionic species of Se(VI) [168]. Based on electrostatic interactions and ion exchange, a speciation scheme for selective determination of Cr(VI) was developed by Dai et al., using chemically modified chitosan [164]. Grafting 2-hydroxyethyltrimethyl ammonium chloride onto the biopolymer provided reliable retention of Cr(VI) by the quaternary ammonium groups at pH 4, while Cr(III) was not captured because chelate complex formation at this pH value is less probable. As was mentioned above, being a deacetylated form of chitin, chitosan has numerous hydroxyl and amino groups, which considerably increase the adsorption of metal ions via chelate formation [183,184]. The high complexability of chitosan was used in the preparation and application of composite materials for nonchromatographic speciation analysis of chromium in tap and surface water [165,166]. An excellent separation of Cr(III) and Cr(VI) using chitosan-modified magnetic nanoparticles was reported by Cui et al. [165]. Based on chelating interactions, quantitative sorption of Cr(III) was achieved at pH 9, while the percentage of retained Cr(VI) was below 10%. A significant benefit of this composite is the ability to simultaneously extract both Cr species at pH 6, which allows preconcentration and determination of Cr(III) and total Cr to be easily performed by simply varying pH in accordance with Approach 2. Another chitosan-based nanocomposite for the extraction of Cr(III) was prepared as a film loaded with silver nanoparticles (AgNPs) [166]. The separation of chromium species was accomplished at pH 8.5 via electrostatic attraction between positively charged ammonium complexes of Cr(III) and negatively charged surface of nanocomposite film followed by complexation with functional groups of chitosan. Quantitation of Cr(VI) was done according to the A4 speciation strategy (Figure 1). The role of AgNPs was not only to increase the adsorptive properties of the chitosan film but also to improve the mechanical strength of the sorbent by cross-linking [166]. A method for quantifying silver nanoparticles in environmental water was developed by using magnetic chitosan microspheres as an adsorbent [170]. The selective retention of AgNPs in the presence of Ag^+^ was explained by the polycationic nature of chitosan at pH < 6. Adsorption of AgNPs at pH 4.5 was a result of the electrostatic attraction of the electron-donating ligands (poly(vinyl pyrrolidone (PVP), citrate, and poly(vinyl alcohol) (PVA)) used as stabilizing agents.

#### 2.11.2. Cellulose-Based Materials

Cellulose is the most abundant biopolymer, consisting of D-glucopyranose monomer units linked by β-1,4-glycosidic bonds. Despite all the advantages as a green biosorbent, raw cellulose finds limited utilization in the field of speciation analysis because it is hard to provide the desired selectivity and adsorptive capacity. On the other hand, the numerous hydroxyl groups facilitate various chemical modifications of the cellulose and thus reveal its great potential in solid-phase extraction techniques. An example of the use of microcrystalline cellulose without any previous functionalization was reported for the speciation analysis of Cr [177]. The sorbent was commercially available as a fine powder and used in the extraction procedure as an aqueous suspension. The separation of Cr(III) and Cr(VI) was achieved at pH 7 owing to the retention of cationic Cr(III) species on the negatively charged sorbent surface. It is worth mentioning that increasing ionic strength reduced adsorption efficiency, but this drawback was overcome by adding surfactant (Triton X-100), so the proposed method for Cr speciation coupled dispersive solid-phase microextraction and cloud point extraction. In most cases, the development of elemental speciation protocol was preceded by chemical modification of cellulose via grafting of different molecules, such as L-cysteine [171], dithiocarbamate [172], and titanium dioxide [174], which enabled the capture of one or more chemical species by complex formation. Zawiswa et al. reported the preparation of TiO_2_-modified membrane via in situ formation of titanium dioxide onto cellulose surface and its further usage for speciation analysis of As and Se in drinking water [174]. The excellent selectivity of the sorbent toward As(V) and Se(IV) was explained by surface complexation with TiO_2_. A significant advantage of this method is the EDXRF assay of retained species directly onto the TiO_2_@cellulose membrane, avoiding the elution step. Lukojko et al. worked with the same measurement technique for quantitation of As(III), preliminary sorbed on cellulose membrane coated with silica and functionalized with (3-mercaptopropyl)-trimethoxysilane [178]. The strong selectivity of the sorbent toward As(III) in the presence of As(V) was demonstrated at pH 1 and explained by the ability of soft acids (As(III)) to react with soft bases (thiol groups). Furthermore, the crucial role of mercapto-functionalization was clearly proven by the superior adsorption of the analyte compared to the unmodified membranes. As remarked for the chitosan derivatives, amino-functionalization is a good approach to providing biopolymeric materials with polycationic properties in an acidic medium, which can be easily controlled by a simple pH adjustment. This is a great facility for the separation of oppositely charged chemical forms of elements, taking into account the dependence of species distribution on pH. In such a manner, by the attraction of the anionic Cr(VI) and repulsion of the cationic Cr(III) at pH 4, amino-sillanized cellulose membranes were implemented in a method for speciation analysis of Cr in water samples [173]. Furthermore, three amino-silanes containing different numbers of amino groups were used for the surface modification, and it was undoubtedly confirmed that the adsorption capacity value enhanced proportionately with the number of amino groups.

Polymer inclusion membranes (PIM) based on cellulose triacetate (CTA) and Aliquat 336 were applied for the efficient separation and determination of As(III) and As(V) by AAS [185]. The membrane containing 50% CTA/50% Aliquat 336 ensures complete transfer ofAs(V) from a pH 7 feed solution to a 0.1 M NaCl stripping solution within 5 h. A novel device based on this PIM was constructed and used to separate As(V) and As(III) and the results showed that 99.7 ± 0.2% of As(V) were successfully separated from As(III). The developed method can be used in addition to the determination of the redox potential of ground water and for the assessment of the level of arsenic contamination of ground water.

#### 2.11.3. β-Cyclodextrin, Dextran and Alginates

β-Cyclodextrin is a cyclic heptasaccharide consisting of D-glucopyranose monomeric units linked by α-1,4-glycosidic bonds and forming a conical-shaped structure with a strongly hydrophobic inner cavity and hydrophilic external surface. Similar to the previously discussed biopolymers, β-cyclodextrin also has a large number of hydroxyl groups, which gives an opportunity for different chemical modifications aiming for high adsorptive capacity and selectivity. A cross-linked polymer, obtained through cross-linking of β-cyclodextrin with epichlorohydrin, was used as an adsorbent for selective retention of Cr(III) chelate with 4-(2-pyridylazo)-resorcinol [180]. The solid-phase extraction was conducted in column mode with a preliminary conditioning of the column with buffer solution (pH 6.0). At this pH value, excellent separation of Cr(III) and Cr(VI) was achieved, and the method was applied in the speciation analysis of Cr in tap water samples. Another speciation scheme for the determination of Mn(VII) and Mn(II) was developed with the aid of β-cyclodextrin modified with ionic liquid (α-picoline) and attached to Fe_3_O_4_ nanoparticles [179]. The magnetic nanocomposite demonstrated a high affinity for Mn(VII) in a wide pH range (6–11) since Mn(II) was adsorbed at pH 9–11, and thus, following the A2 speciation scheme, Mn(VII) and total manganese were extracted at pH 6 and pH 10, respectively. The adsorptive capability toward Mn(VII) and Mn(II) was explained by the synergic effects of electrostatic attraction, chemical inclusion, and intermolecular forces.

Dextran is a bacteria-derived polysaccharide consisting of α-1,6 linked d-glucopyranose units with some α-1,2-, α-1,3-, and α-1,4,-linked side chains. Commercially available cross-linked dextran (Sephadex G-150) was involved in the preparation process of a magnetic composite intended for speciation analysis of chromium in water, tea, and coffee [181]. The presence of dextran in the sorbent structure had an essential impact on the selective adsorption of Cr(VI) because it relied on the protonation of the functional groups at low pH values.

**Figure 4:**
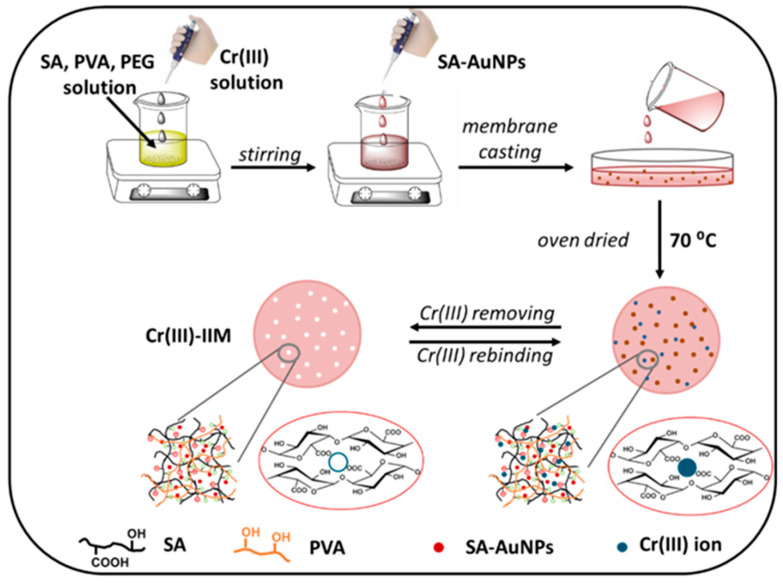
Preparation of Cr(III)-imprinted hydrogel membrane [186].

Based on the ion-imprinting concept, a hydrogel membrane was synthesized via sodium alginate and PVA as film-forming materials, sodium alginate-coated gold nanoparticles as a nontoxic cross-linking and mechanically stabilizing component, and Cr(III) ions as template species [186]. Sodium alginate is a natural anionic polysaccharide composed of 1,4-linked β-D-mannuronic acid and 1,4-linked α-L-guluronic acid residues containing carboxyl and hydroxyl groups. As can be seen from Figure 4, the functional groups of sodium alginate take part in the imprinting of Cr(III) ions, hence, involving this biopolymer in the sorbent composition has a crucial role for the extraction efficiency. As-synthesized membranes were successfully applied in the speciation analysis of Cr following Approach 4 (Figure 1).

#### 2.11.4. Peptides

Peptides are composed of amino acid units connected through amide (peptide) bonds, and in accordance with their structure, they can offer various binding sites for different chemical species. An unconventional approach for speciation analysis of Cr was developed via adsorption of Cr(III) using Cr(III)-binding peptide (YKASLIT), selected through biopanning from phage display peptide library and immobilized onto the surface of microcarrier beads cytopore [182]. It was demonstrated that the Cr(III)-binding phage beads can also adsorb Cr(VI), so preliminary removal of Cr(VI) was required. It was achieved by incubating the sample solution with the cytopore beads, which retained only Cr(VI). In accordance with strategy A1 (Figure 1), total Cr was measured by ICP-MS after the conversion of Cr(VI) to Cr(III) with the aid of ascorbic acid [182].

#### 2.11.5. Analytical Aspects of Biopolymers

**Figure 5:**
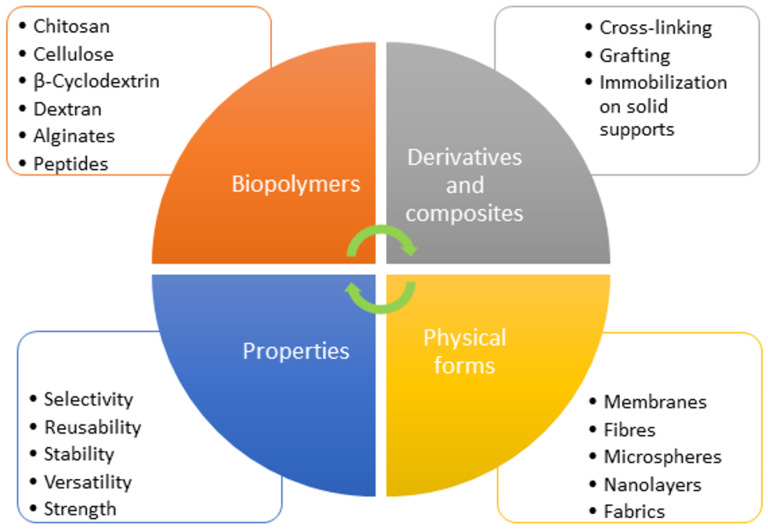
Biopolymeric adsorbents in nonchromatographic speciation analysis.

As was discussed in the previous sections, the biopolymeric materials involved in the elemental speciation assays are mainly derivatives and composite adsorbents, which can be produced in various sizes and shapes, such as fibers [171], membranes [173,174,178,186], microspheres [170], nanolayers [175], films [166], fabrics [176], etc. The chemical functionalization and cross-linking of biopolymers, as well as combining them with miscellaneous nanoparticles, e.g., magnetic [165,170,179,181], silver [165,166], gold [186], and zirconium oxide [175] have aimed to evolve their potential, bringing about higher selectivity and extraction efficiency, reusability, versatility, easy operation, low cost, mechanical strength, and stability (Figure 5).

From an analytical viewpoint, the most frequently used approach is the way of A1 [164,165,168,177,180,181,182]. Following this scheme, various types of water were tested for Cr [164,165,175,180,181,182] and Se [168] species. Another option is the SPE of particular species in parallel samples under different conditions. In this way, chromium (III) and (VI) were selectively extracted using nanozirconium oxide coated with carboxymethyl cellulose nanolayer at pH 7 and pH 2, respectively, and then determined by AAS [175]. A different speciation scheme proposes a combination of selective adsorption of the target species onto membranes and subsequent direct measurement by energy-dispersive X-ray fluorescence (EDXRF) spectrometry with no need for an elution step [173,174,178].

Another aspect of the implementation of biopolymeric materials in speciation analysis is the simultaneous preconcentration of two or more chemical forms, which are subsequently measured with an appropriate analytical technique, ensuring reliable differentiation of individual species. In this way, cellulose fiber grafted with L-cysteine was used as an adsorbent for the simultaneous extraction of Hg(II) and methylmercury at pH 6, followed by AFS measurement in cold atomization mode for Hg(II) and heat/flame atomization mode for total mercury. The concentration of methylmercury was found to be the difference between total and inorganic Hg [171]. In chromium speciation, cellulose fabric modified with a sol–gel/polytetrahydrofuran composite was used for concomitant adsorption of morpholino dithiocarbamate chelates of Cr(III) and Cr(VI), and after elution, species separation and determination were performed by HPLC [176]. A summary of the usage of polymeric biomaterials is presented in Table 5.

## 3. Concluding Remarks and Future Trends

Speciation analysis is undoubtedly one of the most serious challenges facing analytical practice. The different mobility and toxicity of chemical species is well known, and from this point of view, the control and the quality assessment of the environment requires determination of toxic species instead of total element concentrations. The analytical control of environmental samples would be meaningless without the knowledge of the exact concentrations of toxic chemical species. The same is valid for (eco)toxicological implications—toxicity of different chemical species is highly different, and results based on total concentration would not be useful for ecotoxicity assessment.

Procedures involving solid-phase extraction are probably the most widely used for the enrichment of inorganic and organic trace analytes prior to instrumental measurement. In addition, due to the number of advantages, SPE is a preferable procedure for nonchromatographic speciation analysis. The role of the sorbent used is, of course, the most important in terms of extraction efficiency and selectivity. A survey of the literature reveals that polymeric sorbents are widely applied as a result of good opportunities, thanks to their ability to develop efficient, green procedures. In this sense, the design and utilization of biodegradable or reusable polymeric sorbents are very important to minimize waste and reduce the environmental impact of SPE processes. In addition, combining them with convenient measurement techniques that do not require the elution of the analytes can bring the methods significantly closer to the principles of green analytical chemistry. The large diversity of polymeric sorbents with various structures and functionalities makes them a preferred tool for nonchromatographic separations of chemical species not only due to their excellent adsorptive capabilities but also their versatility and tunable selectivity. Synthesis and application of highly selective sorbent materials with large adsorption capacity and good mechanical stability would be the focus of future research works. Their practicality and applicability might be improved by introducing a magnetic core in their structure and incorporating them in a membrane as a support or immobilization in a monolith. Implementation of polymeric composites with multiple modes of interactions (van der Waals, ion exchange, complex formation, etc.) is a good way to achieve broader applicability of polymeric materials in the field of speciation analysis, with a special focus on the concurrent determination of several species with the aid of a single sorbent. Furthermore, the preparation of such multimodal polymeric composites should be designed not only to ensure sufficiently high extraction efficiency and selectivity but also to possess mechanical features allowing on-site analysis as well as easy operation and automation. Beyond any doubt, polymeric sorbents have a significant potential in elemental speciation analysis that will continue to be used and expanded in the future.

## Figures and Tables

**Table 1:** Application of commercially available polymeric materials for elemental speciation by SPE.

Sorbent	Separated Species	pH or Acidity	Sorption Capacity, mg/g	Sample	Analytical Technique	LOD, μg/L	Ref.
Amberlite IRA 900	As(III), As(V)	4.0	229.9	Underground hot water, tap water	ETAAS	- 0.126	[22]
Amberlite IRA 410	As(III), As(V)	7–7.5	13.2	Natural and drinking water	Spectrofluorometric	- 0.75	[25]
Dowex 1X8 SAX resin	As(III), As(V)	6.0–8.0		Groundwater, waterworks	HR-CS-GFAAS and TXRF	0.5	[23]
Dowex 1X8 resin	As(III), As(V)	8 M HCl 8 M acetic acid		NRCC CRM DORM-2, ground water	INAA	20–45	[27]
Dowex 1X8 resin	As(III), As(V)	1 M acetic acid		Ground water	CPAA	10	[28]
717 anion exchange resin	As(III), As(V)			CRM of riverine water (SLRS-4), lake water	HG-AFS	0.02 0.3	[26]
Tetrahydroborate immobilized on Amberlite IRA-400	As(III), As(V)	0.1 M HCl		Tap, well, pond and seawater	FI-HG-AFS	13 ^1^ 15 ^1^	[29]
Lewatit MonoPlus M 500 HY resin	As(III), As(V)	7.50		Drinking water	ICP-MS	0.24	[31]
Lewatit MonoPlus M 500 HY-Fe resin HY-Ag resin	As(III), As(V), DMAs(V), MMAs(V)	9		Drinking, natural and wastewater	ICP-MS or HGAAS	0.2	[30]
Amberlite IRA 910	Cr(III), Cr(VI)	4.5		Seawater	FI-ICP-MS	0.03 0.009	[32]
Lewatit Ionac SR-7	Cr(III), Cr(VI)	3	17	Wastewater, tap water	FAAS	- 0.003	[33]
Dowex-1	Cr(III), Cr(VI)	4.5		Soils, sediments	ICP-OES	- 0.02	[34]
Dowex-1	Cr(III), Cr(VI)	4.5		Black tea, green tea, spinach, fruit trees	ICP-OES	18 ^2^ 21 ^2^	[35]
TSK IC-Cation	Cr(III), Cr(VI)	3.4		Water samples	HPLC-UV-Vis	0.8 ^1^ Cr_total_ 0.6 ^1^ Cr(VI)	[39]
Dowex 21K and poly 2-(5-methylisoxazol)methacrylamide-co-2-acrylamido-2- methyl-1-propane sulfonic acid-co-divinyl-benzene	Cr(III), Cr(VI)	3		Environmental water	FAAS	0.05 0.3	[41]
Amberlite CG-120	Cr(III), Cr(VI)			Spring, drinking and wastewater	FAAS	0.3	[40]
Dowex M-41	Hg(II), methylHg	3% HCl		Estuarine water samples	FIA-HG-QFAAS	1.9 ^3^ 0.8	[36]
Amberlite XAD-7HP	Sb(III), Sb(V)	1.5 M HCl		Meglumine antimoniate	ICP-OES	44, 52	[37]

^1^ ng/L; ^2^ ng/g; ^3^ ng.

**Table 2:** Application of modified and impregnated commercial resins for elemental speciation by SPE.

Sorbent	Separated Species	pH or Acidity	Sorption Capacity, mg/g	Sample	Analytical Technique	LOD, μg/L	Ref.
1,10-Phenanthroline immobilized on Amberlite XAD-16	Cr(III), Cr(VI)	5	33.2	Industrial water	FI-FAAS	0.09	[44]
Xylenol Orange Functionalized Amberlite XAD-16	Cr(III), Cr(VI)	5	26.7	Industrial water	FI-FAAS	0.11	[45]
α-benzoin oxime modified Amberlite XAD-16	Cr(III), Cr(VI)	5	27.6	Industrial water	FI-FAAS	0.14	[46]
Salicylic acid loaded on Amberlite XAD-16	Cr(III), Cr(VI)	5	29.4	Industrial water	FI-FAAS	0.1	[47]
Dithizone modified Dowex Optipore L493	Cr(III), Cr(VI)	5	31.8	Industrial water	FI-FAAS	0.13	[48]
Aminated Amberlite XAD-4	Cr(III), Cr(VI)	8	67	Mineral, drinking and wastewater	MIS-FAAS	0.041	[43]
Dowex M 4195 chelating resin with bis-picolylamine functional group	Cr(III), Cr(VI)	2	29.7	Tap, river and electroplating water	FAAS	- 1.94	[50]
Amberlite XAD-1180 resin	Cr(III), Cr(VI)	0.5 M H_2_SO_4_	-	Food, water, pharmaceutical samples	FAAS	7.7 Cr(VI) 8.6 Cr_total_	[49]
CYPHOS 101 modified Amberlite XAD-1180	Hg(II), methylHg	4 M HCl	-	CRMs (BCR-463 Tuna Fish and Tuna Fish ERM-CE 464); fish samples	ASV	0.40, 0.50 -	[52]
CYPHOS 101 modified Amberlite XAD-1180	Hg(II), methylHg	4 M HCl	-	CRMs (Tuna Fish ERM-CE 464); fish samples	SW-ASV	- 0.45	[53]
CYPHOS 101 modified Amberlite XAD-4, XAD-16 and XAD-1180	Hg(II), methylHg	3 M HCl	-	Water samples	CV-AAS	2.4 ^1^	[55]
Aminated Amberlite XAD-4	Hg(II), methylHg	4.0	-	Fish and mussel samples	FI-CVG-AAS	0.148, 0.157	[42]

^1^ ng/L.

**Table 3:** Application of ion-imprinted polymers for elemental speciation by SPE.

Template Species	Functional Monomer/ Cross-Linker	Separated Species	pH/EF	Capacity, mg/g	Sample	Analytical Technique	LOD, μg/L	Ref.
Chemical immobilization								
As(III)	1-VIA/DVB	As(III), As(V)	8.5/50	-	Fish; CRM (dogfish muscle), dogfish liver, mussel tissue	HPLC-ICP-MS	0.32 ^1^ 0.39 ^1^	[100]
As(III)	1-VIA/DVB	As(III), As(V)	8/10	-	Rice	HPLC-ICP-MS	0.20 ^1^ 0.41 ^1^	[101]
As(V)	ITA/EGDMA	As(III), As(V)	3–4/-	-	Water	TLC/LA-ICP-MS	- 0.06	[104]
ROX	NOBE/TMPTMA	ASA, ROX, NPA, CBA	-/-	1.708 2.040 1.580 1.504	Feeds, edible chicken and pork	HPLC	21.3 ^1^ 41.4 ^1^ 31.7 ^1^ 32.3 ^1^	[102]
Sn(II)	NATU/EGDMA	Sn(II), Sn(IV)	6/-	-	Water, plasma samples	LA-ICP-MS	0.3, 0.4	[105]
Trapping technique								
Sb(III)–APDC complex,	ST/EGDMA	Sb(III), Sb(V)	5/232	6.7	Water, fruit juices	ETAAS	3.9 ^2^	[106]
Sb(III)–thionalide complex	MAA/TMPTMA	Sb(III), Sb(V)	8/25	-	Surface water	ICP-OES	1 ^3^	[107]
Cr(III)–PDC complex	AA/EGDMA	Cr(III), Cr(VI)	3.50–4.75/10	1.3 ^4^	Tap and river water, municipal sewage	ETAAS	0.018	[108]
Cr(III)–8-HQ	ST/DVB	Cr(III), Cr(VI)	9/33	8.5	CRM of wastewater	FAAS	2.1	[109]
Cr(III)–nicotinate complex	AA/EGDMA	Cr(III), Cr(VI)	9–10/-	-	Wastewater	FAAS	0.08 ^5^	[110]
Cr(III)–1,10-phenanthroline complex	ST or ST + 4-VP/EGDMA	Cr(III), Cr(VI)	4.5/20	1.18 ^6^	Tap water and infusion of green tea	ETAAS	0.018	[111]
Fe(II)–BP complex	4-VP/TMPTMA	Fe(II), Fe(III)	7/-	28.01 ^7^	Wine	ETAAS	0.03 Fe_total_, 0.1 Fe(III)	[112]
Fe(II)–BP or PAR complex	4-VP or HEMA/TMPTMA	Fe(II), Fe(III)	7/-	1.79 ^7^	Surface water	ETAAS	0.5, 0.2	[113]
Fe(III)–DHBPT complex	MAA/EGDMA	Fe(II), Fe(III)	4.5/-	40.41	Food, water	FAAS	- 1.56 Fe(III)	[114]
Fe(III)–8-HQ complex	MAA/DVB	Fe(II), Fe(III)	2.5/240	170 ^7^	Drinking water	FAAS	-	[115]
Hg(II)–TAN complex	MAA/TMPTMA	Hg(II), methylHg	7/-	32.0 ^7^	Seawater, mineral water	CV-AAS	0.006	[116]
Hg(II)–PDC complex	MAA/TMPTMA		7/25	64.0 ^7^	River water	CV-AAS	0.015 ^3^, 0.02 ^3^	[117]
Hg(II)–TAN complex	MAA or VIA or HEMA/TMPTMA	Hg(II), methylHg	7/-	73.7 ^7^ (MAA), 78.0 ^7^ (VIA)	Surface water	CV-AAS	0.005, 0.006	[118]
MeHg–phenobarbital complex	MAA/EGDMA	Hg(II), methylHg, ethylHg	8/50	-	Seawater	HPLC-ICP-MS	0.003, 0.002, 0.002	[119]
MeHg–phenobarbital complex	MAA/EGDMA	methylHg, Hg(II)	9/50	-	Seaweed	LC-ICP-MS	0.007 ^1^ 0.02 ^1^	[120,122]
Sn(IV)–PAR complex	MAA/EGDMA	Sn(IV), Sn(II)	8/-		Tap water, river water, food samples	GF-AAS	1.3	[121]
Surface imprinting technique								
As(III)–MAC complex As(V)–MAC complex	HEMA/EGDMA on Fe_3_O_4_ HEMA/EGDMA on Fe_3_O_4_	As(III), As(V)	5/-	76.83 85.57	Water	ICP-MS	-	[129]
Cr(III)	3-aminopropyl-triethoxysilane on SBA-15	Cr(III), Cr(VI)	6/−	38.50	Plating and leather wastewater	ICP-AES and UV-vis	0.53	[126]
Cr(VI)	1-(trimethoxysilylpro-pyl)-3-methylimid-azolium on silica gel	Cr(III), Cr(VI)	2–3/-	6.42 ^7^	Surface water	ETAAS	- 0.02	[125]
Cr(VI)	1-(trimethoxysilyl-propyl)-3-methyl-imidazolium on µSiO_2_	Cr(III), Cr(VI)	3/-	9.4 ^7^	Textiles	ETAAS	- 0.015 ^6^	[124]
Cr(VI)	MAA and 4-VP/MPTMS on SBA-15	Cr(III), Cr(VI)	2/-	96.32	Water	ICP-AES	-	[127]
Cr(VI)	VIA, APTES/MPTMS on Fe_3_O_4_	Cr(III), Cr(VI)	3/98	2.49	Water	FAAS	- 0.29	[131]
Hg(II)–PDC complex	MAA/TMPTMA on silica gel	Hg(II), methylHg	7/-		Wine	CV-AAS	0.02	[127]
Hg(II)–dithizone complex	3-aminopropyltriethoxy-silane	Hg(II), methylHg	6/-		Human hair, fish meat, seawater	AFS	0.015 0.02	[130]
MeHg–PDC complex	MAA/TMPTMA on Fe_3_O_4_@SiO_2_	methylHg, ethylHg, Hg(II)	5/25	25	Natural water	CE-ICP-MS	0.084 ^2^	[132]

^1^ µg/kg; ^2^ ng/L; ^3^ LOQ; ^4^ μg/g; ^5^ μg/mL; ^6^ μg/g; ^7^ μmol/g.

**Table 4:** Metal–organic frameworks in SPE-based methods for speciation analysis.

MOF/Modification	Chemical Species	Sample	EF	Analytical Technique	LOD, ng/L	Ref.
MIL-101(Fe)/dithiocarbamate-modified magnetite nanoparticles	Cr(VI)	Water, tea	470	ETAAS	1	[146]
MIL-101(Cr)/amino dithiocarbamate-modified magnetite nanoparticles	Se(IV)	Water	220	ETAAS	10	[147]
MIL-101(Cr)/1-(2-pyridylazo)-2-naphthol-modified magnetite nanoparticles	Sn(IV)	Water	400	ETAAS	5	[148]
MIL-101(Fe)/morin-modified magnetite nanoparticles	V(V)	Water	400	ETAAS	3	[149]
MIL-101(Cr)/aminodibenzo-18-crown-6 magnetite nanoparticles	Tl(I)	Water	857	ETAAS	1.5	[150]
MOF-199/dithiocarbamate-modified magnetite nanoparticles	As(III)	Water	240	ETAAS	1.2	[151]
MIL-101(Fe) mixed-matrix membrane	As(V)	Water	-	EDXRF	0.094 ^1^	[152]
UiO-66 [Zr_6_O_4_(OH)_4_(bdc)_6_] UiO-66/cysteine-modified	Hg^2+^ phenylHg^+^ methylHg^+^	Water	-	TR-ETAAS	0.06 ^1^ 0.06 ^1^ 0.06 ^1^	[154]
Magnetic Zr–terephtalate MOF composite Magnetic Zr–terephtalate MOF composite/ mercapto-functionalized	As(V) MMAs DMAs As(III)	Squamous carcinoma cells	23 25 24 24	Online MSPME-ICP-MS	4.8 3.8 6.3 7.1	[155]
UiO-66-SH/magnetite nanoparticles	Hg^2+^ methylHg^+^	Water, fish	45.7 47.6	MSPE-HPLC-ICP-MS	1.4 2.6	[156]

^1^ μg/L.

**Table 5:** Applications of biopolymers in SPE-based speciation analysis.

Material/Modification	Chemical Species	Sample	Analytical Technique	LOD, ng/L	Ref.
Chitosan grafted with 2-hydroxyethyltrimethyl ammonium chloride	Cr(VI)	Pond, lake, tap water	FAAS	20	[164]
Chitosan-modified magnetic nanoparticles cross-linked with AgNPs	Cr(III) Total Cr	Environmental water	ICP-OES	0.02 ^1^ 0.03 ^1^	[165]
Chitosan film/loaded with silver nanoparticles	Cr(VI) Cr(III)	Surface water	ICP-MS	0.02 ^2^	[166]
Chitin, Chitosan-immobilized sodium silicate	As(V)	Water	HGAAS ICP-MS	-	[167]
Diethylene Triamine Cross-Linked Chitosan	Se(VI)	Environmental water	ICP-OES	12	[168]
Chitosan-based chelating resin/modified with 3,4,5-trihydroxy benzoic acid	Cr(VI) Cr(III)	-	ICP-AES	-	[169]
Magnetic chitosan microspheres	PVP-AgNPs Cit-AgNPs PVA-AgNPs	Environmental water, wastewater	ICP-MS	0.023 ^2^ 0.016 ^2^ 0.021 ^2^	[170]
Cellulose fibers/modified with L-cysteine	Hg(II) methylHg	Water, cosmetics	CVG-AFS	1 3	[171]
Cellulose/dithiocarbamate-modified	Se(IV) Total Se	Wastewater	LEP-OES		[172]
Cellulose membranes/coated with amorphous silica/amino-silanized	Cr(VI)	Water	EDXRF	0.16 ^1^	[173]
Cellulose membranes/grafted with TiO_2_	Se(IV) As(V)	Water	EDXRF	0.4 ^1^ 0.25 ^1^	[174]
Carboxymethyl cellulose nanolayer/cross-linked using glutaraldehyde onto surface of nano-ZrO_2_	Cr(VI) Cr(III)	Tap and seawater, industrial wastewater	AAS	-	[175]
Cellulose fabric/modified with a sol–gel/polytetrahydrofuran composite	Cr(III) Cr(VI)	Ground and drinking water, wastewater	HPLC-UV	0.001 ^1^ 0.003 ^1^	[176]
Microcrystalline cellulose	Cr(III)	Tap and bottled water	ETAAS	6	[177]
Cellulose membrane/coated with silica/modified with (3-mercaptopropyl)-trimethoxysilane	As(III)	Tap, sea, ground and wastewater	EDXRF	0.045 ^1^	[178]
β-cyclodextrin/modified with ionic liquid/attached on Fe_3_O_4_ nanoparticles	Mn(II) Mn(VII)	Tap, lake and mineral water	ICP-OES	0.15 ^2^ 0.27 ^2^	[179]
β-cyclodextrin cross-linked polymer	Cr(III)	Tap water	GFAAS	0.056 ^2^	[180]
Magnetic dextran (Sephadex G-150)	Cr(VI) Total Cr	Mineral water, Tea, coffee	Voltammetry	0.01 ^3^	[181]
Poly(vinyl alcohol)/sodium alginate/AuNPs hydrogel membranes	Cr(III) Cr(VI)	Water	ETAAS	0.001 ^2^ 0.01 ^2^	[186]
Immobilized Cr(III) binding phages	Cr(III)	Water	ICP-MS	15	[182]

^1^ ng/mL; ^2^ μg/L; ^3^ μM.

## Data Availability

All data are enclosed in this review. Additional information may be supplied on request.

## Abbreviations

AFS	Atomic fluorescence spectrometry
APTES	3-aminopropyltriethoxysilane
APDC	Ammonium pyrrolidinedithiocarbamate
ASA	Arsanilic acid
BP	2,2′-bipyridyl
CBA	Carbarsone
COF	Covalent organic framework
CPAA	Charged particle activation analysis
CRM	Certified reference material
CVG-AFS	Cold vapour generation-atomic fluorescence spectrometry
DBT	Dibutyltin
DDTC	Diethyl dithiocarbamate
DGT	Diffusive gradients in thin films
DHBPT	3,6-bis(3,5-dimethyl-1-H-pyrzol-1-yl)-1,2-dihydro-1,2,4,5-tetrazine
DMAs	Dimethyl arsenic acid
DVB	Divinylbenzene
EDTA	Ethylendiaminetetraacetic acid
EDXRF	Energy-dispersive X-ray fluorescence
EGDMA	Ethylene glycol dimethacrylate
EF	Enrichment factor
ETAAS	Electrothermal-atomic absorption spectrometry
FAAS	Flame-atomic absorption spectrometry
GFAAS	Graphite furnace-atomic absorption spectrometry
HEMA	Hydroxyethyl methacrylate
HGAAS	Hydride generation atomic absorption spectrometry
HG-AFS	Hydride generation atomic fluorescence spectrometry
8-HQ	8-hydroxyquinoline
HPLC	High performance liquid chromatography
iAs	Inrganic arsenic compounds
ICP-MS	Inductively coupled plasma mass spectrometry
ICP-AES	Inductively coupled plasma-atomic emission spectrometry
ICP-OES	Inductively coupled plasma-optical emission spectrometry
IIP	Ion-imprinted polymer
IIP-SPE	Solid-phase extraction with ion-imprinted polymer
INAA	Instrumental neutron activation analysis
ITA	Itaconic acid
LAICP-MS	Laser ablation inductively coupled plasma mass spectrometry
LC	Liquid chromatography
LEP-OES	Liquid electrode plasma-optical emission spectrometry
LOD	Limit of detection
LOQ	Limit of quantification
MAA	Methacrylic acid
MAC	N-methacryloyl-l-cysteine
γ-MAPS	Methacryloxypropyltrimethoxysilane
MBT	Monobutyltin
MMAs	Monomethyl arsenic acid
MOF	Metal-organic framework
NATU	N-allylthiourea
NOBE	N,O-bismethacryl ethanolamine
NPA	Nitarsone
NPs	Nanoparticles
oAs	Organoarsenic compounds
PAR	4-(2-pyridylazo)resorcinol
PDC	Pyrrolidinedithiocarbamate
PEG	Poly(ethylene glycol)
PVA	Poly(vinyl alcohol)
PVP	Poly(vinyl pyrrolidone)
RAFT	Reversible addition-chain fragmentation polymerization
ROX	Oxarsone
SA	Sodium alginate
SBAE	Strong base anion exchange
SPE	Solid-phase extraction
SPME	Solid-phase microextraction
ST	Styrene
TAN	1-(2-thiazolylazo)-2-naphthol
TBT	Tributyltin
TLC	Thin layer chromatography
TMPTMA	Trimethylolpropane trimethacrylate
1-VIA	1-vinyl imidazole
4-VP	4-vinylphiridine
